# A Ferredoxin- and F_420_H_2_-Dependent, Electron-Bifurcating, Heterodisulfide Reductase with Homologs in the Domains *Bacteria* and *Archaea*

**DOI:** 10.1128/mBio.02285-16

**Published:** 2017-02-07

**Authors:** Zhen Yan, Mingyu Wang, James G. Ferry

**Affiliations:** Department of Biochemistry and Molecular Biology, Pennsylvania State University, University Park, Pennsylvania, USA; University of Hawaii—Manoa

## Abstract

Heterodisulfide reductases (Hdr) of the HdrABC class are ancient enzymes and a component of the anaerobic core belonging to the prokaryotic common ancestor. The ancient origin is consistent with the widespread occurrence of genes encoding putative HdrABC homologs in metabolically diverse prokaryotes predicting diverse physiological functions; however, only one HdrABC has been characterized and that was from a narrow metabolic group of obligate CO_2_-reducing methanogenic anaerobes (methanogens) from the domain *Archaea*. Here we report the biochemical characterization of an HdrABC homolog (HdrA2B2C2) from the acetate-utilizing methanogen *Methanosarcina acetivorans* with unusual properties structurally and functionally distinct from the only other HdrABC characterized. Homologs of the HdrA2B2C2 archetype are present in phylogenetically and metabolically diverse species from the domains *Bacteria* and *Archaea*. The expression of the individual HdrA2, HdrB2, and HdrB2C2 enzymes in *Escherichia coli*, and reconstitution of an active HdrA2B2C2 complex, revealed an intersubunit electron transport pathway dependent on ferredoxin or coenzyme F_420_ (F_420_H_2_) as an electron donor. Remarkably, HdrA2B2C2 couples the previously unknown endergonic oxidation of F_420_H_2_ and reduction of ferredoxin with the exergonic oxidation of F_420_H_2_ and reduction of the heterodisulfide of coenzyme M and coenzyme B (CoMS-SCoB). The unique electron bifurcation predicts a role for HdrA2B2C2 in Fe(III)-dependent anaerobic methane oxidation (ANME) by *M. acetivorans* and uncultured species from ANME environments. HdrA2B2C2, ubiquitous in acetotrophic methanogens, was shown to participate in electron transfer during acetotrophic growth of *M. acetivorans* and proposed to be essential for growth in the environment when acetate is limiting.

## IMPORTANCE

Discovery of the archetype HdrA2B2C2 heterodisulfide reductase with categorically unique properties extends the understanding of this ancient family beyond CO_2_-reducing methanogens to include diverse prokaryotes from the domains *Bacteria* and *Archaea*. The unprecedented coenzyme F_420_-dependent electron bifurcation, an emerging fundamental principle of energy conservation, predicts a role for HdrA2B2C2 in diverse metabolisms, including anaerobic CH_4_-oxidizing pathways. The results document an electron transport role for HdrA2B2C2 in acetate-utilizing methanogens responsible for at least two-thirds of the methane produced in Earth’s biosphere. The previously unavailable heterologous production of individual subunits and the reconstitution of HdrA2B2C2 with activity have provided an understanding of intersubunit electron transfer in the HdrABC class and a platform for investigating the principles of electron bifurcation.

## INTRODUCTION

Heterodisulfide reductase (Hdr) was first discovered in CH_4_-producing species (methanogens) from the domain *Archaea* where one or the other of two classes (HdrABC or HdrDE) is essential for all methanogenic pathways (1). However, the genomes of diverse species in the domains *Bacteria* and *Archaea* are annotated with genes encoding HdrABC homologs, suggesting that these genes play roles in a greater diversity of energy-conserving metabolisms, which include the oxidation of methanol and inorganic sulfur compounds (2–4), the reduction of sulfate and ferric iron (4–6), syntrophic utilization of fatty acids (7), and the anaerobic oxidation of CH_4_ (8–10). Indeed, the HdrABC class belongs to the core repertoire of the ancient prokaryotic common ancestor consistent with diverse physiological functions of extant species (11).

Although of ancient origin and widespread, the biochemical and physiological understanding of the HdrABC class is restricted to one homolog essential for the pathway of CO_2_ reduction to CH_4_. The final step in all methanogenic pathways (equation 1) is the reductive demethylation of methyl coenzyme M (CH_3_-SCoM) for which coenzyme B (HSCoB) supplies reductant. The heterodisulfide product is reduced by Hdr (equation 2), releasing HSCoB and HSCoM for methylation.

(1) $${\text{CH}}_{\text{3}}\text{-SCoM + HSCoB}\to {\text{ CH}}_{\text{4}}\text{ + CoMS-SCoB}$$

(2) $$\text{CoMS-SCoB}+{\text{2e}}^{-}\;+{\text{2H}}^{+}\;\to \text{ HSCoM}+\text{HSCoB}$$

The cytoplasmic HdrABC in H_2_-oxidizing CO_2_-reducing species is complexed with the MvhAGD hydrogenase. Electron pairs donated from the hydrogenase are bifurcated by HdrABC, reducing CoMS-SCoB and ferredoxin (Fdx), and Fdx donates electrons for the first step in the reduction of CO_2_ to produce CH_3_-SCoM. The bifurcation is a flavin-based coupling of the endergonic reduction of Fdx with the exergonic reduction of CoMS-SCoB. Flavin-based electron bifurcation is considered to be an ancient energy-conserving mechanism (12–14). Importantly, a comprehensive mechanistic understanding of the HdrABC class has been impeded by the unavailability of a recombinantly produced enzyme or an activity assay utilizing a physiological electron donor. Although the obligate two-electron carrier coenzyme F_420_ (F_420_) has diverse roles in species from the domains *Bacteria* and *Archaea*, roles in electron bifurcation and the donor to HdrABC homologs are still unknown (15).

More-diverse roles are postulated for electron bifurcation catalyzed by HdrABC homologs in phylogenetically and physiologically diverse species in the domains *Bacteria* and *Archaea* (12, 13, 16). Bioinformatic analyses of methanogens and nonmethanogens from the domains *Bacteria* and *Archaea* predict an HdrABC homolog wherein the HdrA subunit is fused with MvhD (see Fig. S1 in the supplemental material). Genes encoding the homolog, designated HdrA2B2C2, are present in the methanogenic archaeon *Methanosarcina acetivorans* with metabolic capabilities distinct from those of obligate CO_2_-reducing methanogens (17, 18). The fused HdrA2 subunit and low sequence identity of all subunits with subunits of the HdrABC homolog from obligate CO_2_-reducing methanogens (29 to 37%) predict differences in structure and function (19).

10.1128/mBio.02285-16.2 FIG S1  Sequence alignments of HdrA2 from *M. acetivorans* with homologs. The MvhD domain sequences are shown in boldface type. Cysteine residues conserved in HdrA (CAA57039) and MvhD (AAB02349.1) from *Methanothermobacter thermautotrophicus* are marked with an asterisk, whereas others are marked with a pound symbol. Alignments were made with the COBALT multiple alignment tool. The sequences belong to the following species (the corresponding percent coverage and identity to *M. acetivorans* HdrA2 are shown before and after the slash, respectively, in parentheses): WP_011022820.1, *Methanosarcina acetivorans* (100/100); WP_048037482.1, *Methanosarcina mazei* (100/95); WP_011307542.1, *Methanosarcina barkeri* (98/94); WP_023843920.1, *Methanolobus tindarius* (98/67); WP_015324480.1, *Methanomethylovorans hollandica* (98/67); WP_048194718.1, *Methanococcoides methylutens* (98/66); WP_013897960.1, *Methanosalsum zhilinae* (98/65); WP_013194860.1, *Methanohalobium evestigatum* (98/65); WP_013037418.1, *Methanohalophilus mahii* (98/65); WP_048089472.1, “*Candidatus* Methanoperedens  nitroreducens” (98/61); WP_014586170.1, *Methanosaeta harundinacea* (98/61); WP_042684393.1, *Methermicoccus shengliensis* (98/55); WP_012964674.1, *Ferroglobus placidus* (98/53); WP_010878733.1, *Archaeoglobus fulgidus* (97/55); KPL16356.1, *Bacteroides* sp. strain SM23_62 (97/48); KPV63986.1, “*Candidatus* Bathyarchaeota archaeon” BA1 (96/49); WP_051309217.1, *Desulfobulbus japonicas* (96/47); WP_041286569.1, *Desulfomonile tiedjei* (97/45); KPJ88130.1, *Spirochaetes* bacterium DG_61 (97/46). Download FIG S1, DOCX file, 0.02 MB. Copyright © 2017 Yan et al. 2017 Yan et al. This content is distributed under the terms of the Creative Commons Attribution 4.0 International license.

Here we report the individual heterologous production and characterization of HdrA2, HdrB2, and HdrB2C2 from *M. acetivorans* that form an HdrA2B2C2 complex with Fdx^2−^- and F_420_H_2_-dependent heterodisulfide reductase activity. The experimental approach has advanced an understanding of intersubunit electron transfer for the HdrABC class and uncovered a previously unknown coenzyme F_420_H_2_-dependent electron bifurcation that predicts roles for HdrA2B2C2 homologs in pathways of anaerobic CH_4_ oxidation in *M. acetivorans* and other species (20–22). The uncommon properties of HdrA2B2C2 identify it as representative of an HdrABC subclass with homologs in diverse species from the domains *Bacteria* and *Archaea*.

## RESULTS

### Bioinformatic analyses.

A search of the nonredundant databases (https://www.ncbi.nlm.nih.gov/pubmed) with HdrA2 as the query retrieved 150 sequences, of which 77 were HdrA2 homologs containing HdrA and MvhD domains with greater than 46% identity and 96% coverage. Figure S1 in the supplemental material shows an alignment of representative sequences. A total of 48 HdrA2 homologs were from acetotrophic and methylotrophic methanogens. None of the homologs were from obligate CO_2_-reducing methanogens, indicating a role for HdrA2 specific to acetotrophic and methylotrophic methanogens. Of the 77 HdrA2 homologs, 29 were from physiologically and phylogenetically diverse nonmethanogenic species in the domains *Bacteria* and *Archaea*. Of the 150 sequences retrieved, 73 were homologs of the canonical HdrA from both methanogenic and nonmethanogenic species. These results indicate that HdrA2 homologs play roles in metabolically diverse species, warranting investigation of an HdrA2 representative chosen from *M. acetivorans* that grows by converting acetate to CH_4_ (17).

### Recombinant Fdx.

**FIG 1:**
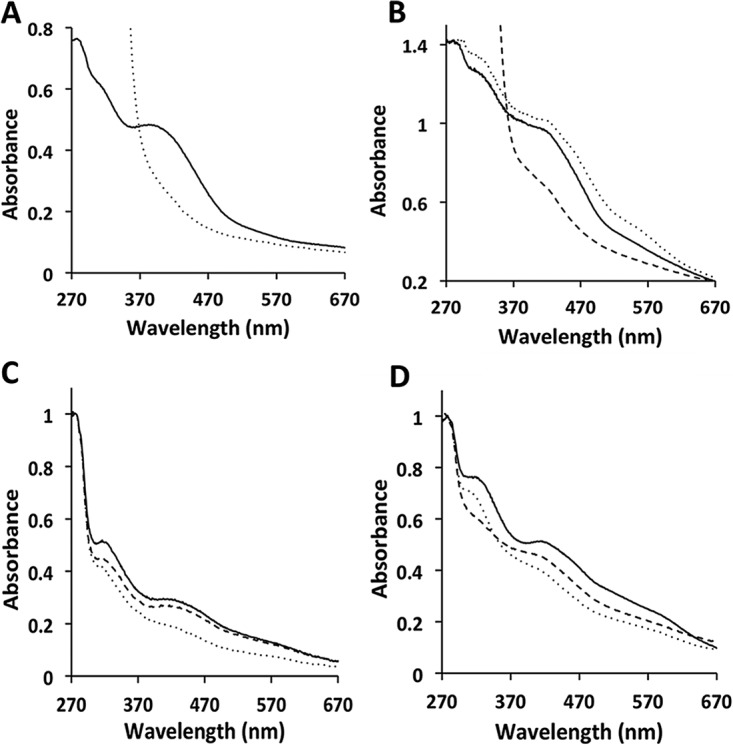
UV-Vis spectra of recombinantly produced ferredoxin (Fdx), HdrA2, HdrB2, and HdrB2C2 from *M. acetivorans*. All proteins were contained in 50 mM Tris buffer (pH 8.0). Spectra were recorded with a Cary 50 Bio UV-Vis spectrophotometer. (A) Spectra of 15.8 μM Fdx. Spectra of Fdx as purified (solid line) and as purified and reduced with sodium dithionite (dotted line) are shown. The CODH/ACS purified from acetate-grown *M. acetivorans* reduced 33.3 μM Fdx at a rate of 55.3 nmol/min/mg CODH/ACS. (B) Spectra of 12.2 µM HdrA2. Spectra of HdrA2 as purified (solid line), reconstituted with FAD (dotted line), reconstituted with FAD and reduced with sodium dithionite (dashed line). (C) Spectra of 18.3 µM HdrB2. Spectra of HdrB2 as purified (solid line), reduced with 0.1 mM sodium dithionite (dotted line), and the reduced protein after the addition of 0.1 mM CoMS-SCoB (dashed line). (D) Spectra of 11.2 µM HdrB2C2. Spectra of HdrB2C2 as purified (solid line), reduced with 0.1 mM sodium dithionite (dotted line), and the reduced protein after the addition of 0.1 mM CoMS-SCoB (dashed line).

The conversion of acetate to CH_4_ begins with activation to acetyl coenzyme A (acetyl-CoA) that is cleaved at the C-C and C-S bonds producing methyl and carbonyl groups catalyzed by CO dehydrogenase/acetyl-CoA synthase (CODH/ACS) (23, 24). The methyl group is transferred to HSCoM, and the carbonyl group is oxidized to CO_2_ with reduction of Fdx. Thus, Fdx is a candidate electron donor/acceptor of HdrA2B2C2. Of multiple Fdx-encoding genes, the Fdx encoded by MA0431 is produced in acetate-grown *M. acetivorans* and is the electron acceptor for CODH/ACS (25). The Fdx produced in *Escherichia coli* strain BL21(DE3) Δ*iscR* (Fig. 1A) showed a prominent absorption band centered at ~390 nm with an *A*_390_/*A*_280_ ratio of 0.69, indicating a nearly full complement of the predicted two [Fe_4_S_4_] clusters (25). The recombinant Fdx was competent in accepting electrons from CODH/ACS at a rate of 55.3 nmol/min/mg.

### Recombinant HdrA2, HdrB2, HdrC2, and HdrB2C2.

HdrA2, HdrB2, HdrC2, and HdrB2C2 were produced in *E. coli* strain BL21(DE3) Δ*iscR*. All except HdrC2 were present in the cytoplasm and purified to homogeneity (Fig. S2). HdrC2 was present in inclusion bodies and not purified. When coproduced with HdrB2, HdrC2 was present in the cytoplasm and copurified with HdrB2 to homogeneity (Fig. S2). The results indicate that either HdrC2 was misfolded or the presence of HdrB2 is required for HdrC2 to fold properly. Regardless, the results indicate that HdrB2 and HdrC2 form an HdrB2C2 complex.

10.1128/mBio.02285-16.3 FIG S2  SDS-PAGE of purified recombinant proteins produced in *Escherichia coli* and CODH/ACS purified from *Methanosarcina acetivorans*. The Bolt 4 to 12% gels (Invitrogen) were stained with Coomassie brilliant blue. The numbers at the sides of the gels refer to the molecular masses (in kilodaltons) of adjacent standards. Amounts loaded: HdrA2 and HdrB2 (10 μg); HdrB2C2 and CODH/ACS (25 μg). Download FIG S2, DOCX file, 0.2 MB. Copyright © 2017 Yan et al. 2017 Yan et al. This content is distributed under the terms of the Creative Commons Attribution 4.0 International license.

HdrA2 migrated in SDS-polyacrylamide gels with an apparent molecular mass of 87 kDa (Fig. S2) consistent with the molecular mass of 86.9 kDa calculated for the HdrA-MvhD fusion. The UV-visible (UV-Vis) spectrum of the protein as purified (as-purified protein) shows prominent absorption bands centered at ~320 and ~420 nm attributed to Fe-S clusters (Fig. 1B). Reconstitution of the as-purified protein with flavin adenine dinucleotide (FAD) resulted in the enhancement of an absorption band centered at ~560 nm attributed to flavin. Indeed, upon reconstitution, the flavin content increased from 0.32 ± 0.06 (*n* = 3) to 1.2 ± 0.1 (*n* = 3) mol per mol of HdrA2. The FAD-reconstituted preparation had 20.6 ± 2.4 atoms of nonheme iron and 18.6 ± 1.3 atoms of acid-labile sulfur per molecule of HdrA2. The ε_420_ was found to be 82.3 mM^−1 ^cm^−1^. These results are consistent with each molecule of HdrA2 containing one FAD molecule and four Fe_4_S_4_ clusters in the HdrA domain, and one Fe_2_S_2_ cluster in the MvhD domain, predicted by motifs in the deduced sequence (Fig. S3).

10.1128/mBio.02285-16.4 FIG S3  Sequence alignments of HdrA2B2C2 from *Methanosarcina acetivorans* with HdrABC and MvhD of *Methanothermobacter thermautotrophicus*. (A) M.t. HdrA, HdrA from *M. thermautotrophicus* (CAA57039); M.a. HdrA2, HdrA2 from *M. acetivorans* (AAM06247.1); M.t. MvhD, MvhD from *M. thermautotrophicus* (AAB02349.1). The overall sequences of HdrA and MvhD were found to be 50 and 49% identical with the corresponding domains in HdrA2. The sequence comparisons revealed four Fe_4_S_4_-binding motifs (CX_2_CX_2_CX_3_CP) common to HdrA of *M. thermautotrophicus* and the HdrA domain (residues 1 to 649) of HdrA2. A motif (CX_2_CX_25_CX_24_CX_4_C) predicted to ligate the Fe_2_S_2_ cluster in MvhD from *M. thermautotrophicus* (62) was found to be conserved in the MvhD domain of *M. acetivorans* HdrA2 (residues 650 to 793). (B) M.t. HdrB, HdrB *M. thermautotrophicus* (CAA57038.1); M.a. HdrB2, HdrB2 from *M. acetivorans* (AAM07582.1). The HdrB2 sequence has 55% identity with HdrB and contains two conserved motifs (CX_31–39_CCX_35–36_CX_2_C) of which one is predicted to ligate the active site [Fe_4_S_4_]^3+^ cluster in obligate CO_2_-reducing methanogens. (C) M.a. HdrC2, HdrC2 from *M. acetivorans* (AAM07581.1); M.t. HdrC, HdrC from *M. thermautotrophicus* (AAB86344.1). The sequence of HdrC2 has 43% identity with HdrC. The comparison shows two Fe_4_S_4_-binding motifs of HdrC2 conserved in HdrC from *M. thermautotrophicus*. Alignments were made with COBALT. Download FIG S3, DOCX file, 0.01 MB. Copyright © 2017 Yan et al. 2017 Yan et al. This content is distributed under the terms of the Creative Commons Attribution 4.0 International license.

HdrB2 migrated in SDS-polyacrylamide gels with an apparent molecular mass of 33 kDa in agreement with a calculated molecular mass of 32.8 kDa (Fig. S2). The UV-Vis spectrum of as-purified preparations showed absorption bands centered at ~320, ~420, and ~580 nm (Fig. 1C). The protein contained 5.0 ± 0.1 (*n* = 3) nonheme iron atoms and 5.1 ± 0.2 (*n* = 3) acid-labile sulfur atoms per molecule of HdrB2. The ε_420_ was found to be 15.9 mM^−1^ cm^−1^. These results are consistent with the presence of an Fe_4_S_4_ cluster ligated by one of two cysteine-rich motifs (CX_31–39_CCX_35–36_CX_2_C) (Fig. S3) that are conserved in HdrB of obligate CO_2_-reducing methanogens and proposed to ligate a novel active site [Fe_4_S_4_]^3+^ cluster (26). The addition of CoMS-SCoB to dithionite-reduced HdrB2 increased the absorbance at ~320 and ~420 nm, indicating that the protein was competent to reduce the heterodisulfide to HSCoM and HSCoB (Fig. 1C). In an apparent anomaly, no significant forward activity (reduction of CoMS-SCoB with reduced methyl viologen [MV]) was detected, either with HdrB2 alone or in combination with HdrA2 and Fdx. However, reverse activity with HdrB2 alone was measurable, and there was no significant change in the *V*_max_ when HdrA2 was present (Table 1). To our knowledge, this is the first heterologously produced and catalytically active HdrB from any source.

**TABLE 1:** Kinetic constants of forward and reverse heterodisulfide reductase activities for combinations of HdrA2, HdrB2, and HdrB2C2

Subunit(s)	Heterodisulfide reductase activity	*V*_max_ (μmol/min/mg)	*K*_*m*_ (mM)
HdrB2C2	Reverse	0.6 ± 0.1	1.4 ± 0.2 HSCoM
			1.2 ± 0.2 HSCoB
HdrB2C2 HdrA2	Forward	2.2 ± 0.3	0.05 ± 0.01 CoMS-SCoB
		4.2 ± 0.2^a^	
	Reverse	0.63 ± 0.1^b^	ND^c^
HdrB2	Reverse	0.5 ± 0.1	1.2 ± 0.3 HSCoM
			1.1 ± 0.2 HSCoB
HdrB2 HdrA2	Reverse	0.6 ± 0.1^b^	ND

^a^ Ferredoxin (50 μg) was added to the reaction mixture for forward activity.

^b^ Determined with saturating amounts (5 × *K*_*m*_) of HSCoM and HSCoB.

^c^ ND, not determined.

The HdrC2 of purified HdrB2C2 migrated in SDS-polyacrylamide gels with an apparent molecular mass of 18 kDa in agreement with a calculated molecular mass of 18.1 kDa (Fig. S2). The UV-Vis spectrum of as-purified HdrB2C2 showed absorption bands centered at ~320, ~420, and ~580 nm (Fig. 1D). Preparations contained 14.6 ± 1.0 (*n* = 3) nonheme iron atoms and 11.9 ± 0.8 (*n* = 3) acid-labile sulfur atoms per molecule of HdrB2C2. The ε_420_ was found to be 45.7 mM^−1^ cm^−1^. These results are consistent with the presence of three Fe_4_S_4_ clusters in the HdrB2C2 complex which is predicted from results obtained for HdrB2 in addition to the sequence of HdrC2 that contains two canonical Fe_4_S_4_-binding motifs (Fig. S3). Thus, the results indicate that as-purified HdrB2C2 had a full complement of iron-sulfur centers.

The addition of CoMS-SCoB to dithionite-reduced HdrB2C2 increased the absorbance at ~420 nm, indicating that the complex was active in reducing the heterodisulfide to HSCoM and HSCoB (Fig. 1D). No significant forward activity was detected unless HdrA2 was present, a result indicating that HdrB2C2 is incapable of accepting electrons from MV and that HdrA2 mediates electron transfer between MV and HdrB2C2. Kinetic analyses (Table 1) showed that *V*_max_ nearly doubled in the presence of both HdrA2 and Fdx. These results, and the finding that HdrB2 in combination with HdrA2 was incapable of catalyzing MV-dependent activity, is consistent with a role for HdrC2 in mediating electron transfer from HdrA2 to HdrB2. Kinetic constants for the reverse activity of HdrB2C2 (Table 1) were not significantly different from those determined for HdrB2, which indicates that HdrC2 plays no role in catalysis by HdrB2.

### Reconstitution of an active HdrA2B2C2 complex.

**FIG 2:**
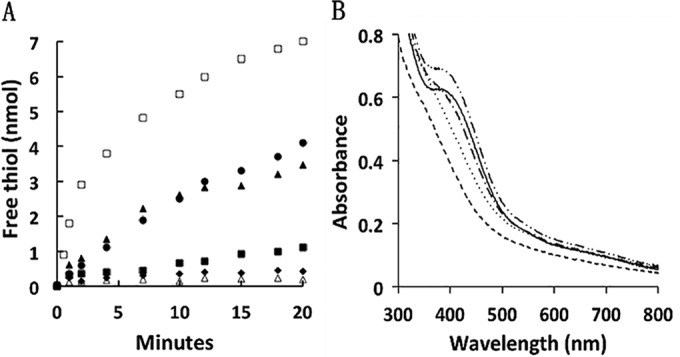
Reconstitution of a system catalyzing the CO-dependent reduction of CoMS-SCoB. (A) Time course of free thiol production from CoMS-SCoB dependent on combinations of HdrA2, HdrB2, and HdrB2C2. The complete reaction mixture (CRM) (0.5 ml) contained 0.1 mg CODH/ACS, 1.5 μM Fdx, 0.8 μM HdrA2, 1.1 μM HdrB2C2 or HdrB2, and 0.1 mM CoMS-SCoB in 50 mM MOPS buffer (pH 7.0). The atmosphere was 100% CO, and the temperature was 20°C. Reactions were initiated by the addition of 0.1 mg CODH/ACS. Initial rates are shown in parentheses where applicable. Time course of free thiol production from CoMS-SCoB in CRM with HdrB2 (0.73 mol/min/mol HdrB2) (▲), CRM with HdrB2 minus HdrA2 (■), CRM with HdrB2C2 (3.6 mol/min/mol HdrB2C2) (□), CRM with HdrB2C2 minus HdrA2 (0.62 mol/min/mol HdrB2C2) (•), CRM minus HdrB2 or HdrB2C2 (⧫), or CRM with HdrB2C2 or HdrB2 minus Fdx (Δ). All activities were dependent on the presence of CODH/ACS and CoMS-SCoB (not shown). (B) Oxidation of ferredoxin catalyzed by HdrA2B2C2 in the presence of CoMS-SCoB. The basal reaction mixture (0.5 ml) contained 9.4 nmol recombinant Fdx and 0.1 mg CODH/ACS in 50 mM MOPS buffer (pH 7.0) with an Ar atmosphere. Alterations were made to the basal mixture in the following order: none (solid line), CO was added to reduce Fdx (dashed line), CO was replaced with Ar and 0.5 μM HdrA2 plus 0.8 μM HdrB2C2 were added (dotted line), 0.1 mM CoMS-SCoB was added (dashed and dotted line), the reaction mixture was exposed to air (dashed and double-dotted line).

Figure 2A shows representative time courses for reduction of CoMS-SCoB catalyzed by various combinations of HdrA2, HdrB2, and HdrB2C2 in the presence of CO, CODH/ACS, and Fdx as electron donors. Substantial initial rates were observed only with HdrB2C2, which indicates that Fdx mediates direct electron transfer from CODH/ACS to HdrB2C2. Rates with only HdrB2 were inconsequential, indicating a role for HdrC2 in the transfer of electrons from Fdx to HdrB2. Rates of Fdx-dependent reduction of HdrB2 or HdrB2C2 (Fig. S4) showed that HdrB2C2 was reduced at a rate fivefold greater than for HdrB2, providing further support for the role of HdrC2 in mediating electron transfer from Fdx to HdrB2. In contrast, the combination of HdrA2 and HdrB2 enhanced the initial rate of CoMS-SCoB to levels comparable with only HdrB2C2 (Fig. 2A). This result indicates that HdrA2 mediates electron transfer from Fdx to catalytic HdrB2 without HdrC2 participation. Reaction mixtures containing a combination of HdrA2 and HdrB2C2 catalyzed the reduction of CoMS-SCoB at initial rates approximately sixfold greater than the rate observed for the combination of HdrA2 and HdrB2 (Fig. 2A). These results indicate that HdrC2 mediates electron transfer from HdrA2 to HdrB2 at a rate substantially greater than the transfer of electrons from HdrA2 directly to HdrB2. Although *hdrA2* (MA2868) is located distant from *hdrB2* and *hdrC2* (MA4237-MA4236), the results establish formation of an HdrA2B2C2 complex catalyzing the most efficient oxidation of Fdx and reduction of CoMS-SCoB. Nevertheless, gel filtration column chromatography of the reconstituted HdrA2B2C2 resulted in separation of HdrA2 from HdrB2C2, indicating a weakly bound catalytic complex.

10.1128/mBio.02285-16.5 FIG S4  Fdx-dependent reduction of HdrA2, HdrB2C2, and HdrB2. It was previously shown that Fdx is an electron acceptor of CO-reduced CODH/ACS (25) that was used to evaluate the ability of recombinant Fdx to reduce HdrA2, HdrB2, and HdrB2C2. The atmosphere was 100% CO, and the temperature was 20°C. Reduction of HdrA2 and HdrB2C2 was monitored at 410 nm with a Cary 50 Bio UV-Vis spectrophotometer. (A) HdrA2 reduction. The reaction mixtures (0.5 ml) contained 0.1 mg CODH/ACS, 15 μM HdrA2, and the indicated concentrations of Fdx in 50 mM MOPS buffer (pH 7.0). Reactions were initiated by the addition of HdrA2 after preincubation of the reaction mixture with CO to fully reduce Fdx. Key to curves or traces: a, minus Fdx; b, 2.2 μM Fdx; c, 4.4 μM Fdx (2.3 nmol HdrA2 reduced/min). (B) HdrB2C2 and HdrB2 reduction. The reaction mixtures (0.5 ml) contained 0.1 mg CODH/ACS, 22.8 μM HdrB2C2 or HdrB2, and the indicated concentrations of Fdx in 50 mM MOPS buffer (pH 7.0). Reactions were initiated by the addition of HdrB2C2 or HdrB2 after preincubation of the reaction mixtures with CO to fully reduce Fdx. Key to curves or traces: a, reaction mixture containing either HdrB2C2 or HdrB2 minus Fdx; b, reaction mixture containing HdrB2 and 4.4 μM Fdx (0.3 nmol HdrB2 reduced/min); c, reaction mixture containing HdrB2C2 and 2.2 μM Fdx; d, reaction mixture containing HdrB2C2 and 4.4 μM Fdx (1.5 nmol HdrB2C2 reduced/min). Comparison of traces b and d indicates that HdrC2 is important for maximizing electron transfer from Fdx to the catalytic HdrB2. Download FIG S4, DOCX file, 0.1 MB. Copyright © 2017 Yan et al. 2017 Yan et al. This content is distributed under the terms of the Creative Commons Attribution 4.0 International license.

The redox potential for the CO/CO_2_ couple (−558 mV) predicts two-electron reduction of Fdx (−520 mV) by CODH/ACS (27). Indeed, results shown in Fig. 2B confirm reduction to Fdx^2−^ in the presence of CODH/ACS and 1.0 atm of CO. However, Fdx^2−^ was only partially oxidized by HdrA2B2C2 in the presence of CoMS-SCoB. A total of 10.1 ± 1.1 (*n* = 3) nmol of free thiols were produced from CoMS-SCoB in the reaction mixture, which contained 9.4 nmol Fdx^2−^. The results indicate that the physiologically relevant reduction of CoMS-SCoB catalyzed by CODH/ACS and HdrA2B2C2 proceeds according to equations 3 and 4 that are summed in equation 5.

(3) $$\text{CO}+2{\text{Fdx}}^{1-}+{\text{H}}_{2}\text{O}\to {\text{CO}}_{2}+2{\text{Fdx}}^{2-}+2{\text{H}}^{+}$$

(4) $$2{\text{Fdx}}^{2-}+2{\text{H}}^{+}+\text{CoMs-SCoB}\to \text{HSCoM}+\text{HSCoB}+2{\text{FDx}}^{1-}$$

(5) $$\text{CO}+{\text{H}}_{2}\text{O}+\text{CoMS-SCoB}\to \text{HSCoM}+\text{HSCoB}+{\text{CO}}_{2}$$

### Interaction of F_420_ with HdrA2 and HdrA2B2C2.

F_420_ is an electron carrier with a multitude of functions in methanogenic and nonmethanogenic species in the domains *Bacteria* and *Archaea* that also contain genes encoding HdrA2B2C2 (15). F_420_ is an obligatory two-electron carrier that requires Fdx:F_420_ oxidoreductases to contain a flavin that accepts an electron from Fdx and generates the hydride for transfer to F_420_. Thus, the flavin-containing HdrA2 is a candidate for interacting with F_420_. Indeed, HdrA2 catalyzed the Fdx-dependent reduction of F_420_ with a *K*_*m*_ for F_420_ of 6.4 µM (Fig. S5), identifying a novel function for heterodisulfide reductases. NAD did not substitute for F_420_, although an HdrA homolog from the acetogen *Moorella thermoacetica* reduces the artificial electron acceptor benzyl viologen with NADH (28).

10.1128/mBio.02285-16.6 FIG S5  Fdx:F_420_ oxidoreductase activity of HdrA2. The atmosphere was 100% CO, and the temperature was 20°C. Reduction of F_420_ was monitored at 420 nm with a Cary 50 Bio UV-Vis spectrophotometer. The reactions were initiated by the addition of F_420_ after preincubation with CO to fully reduce Fdx and HdrA2. (A) Fdx- and HdrA2-dependent reduction of coenzyme F_420_. The reaction mixtures (0.5 ml) contained 0.1 mg CODH/ACS, 2.2 μM Fdx, 15 μM F_420_, and the indicated concentrations of HdrA2 in 50 mM MOPS buffer (pH 7.0). Key to curves or traces: (a) minus HdrA2; (b) minus Fdx; (c) 0.8 μM HdrA2 (5.1 nmol of F_420_ reduced/min/mg of HdrA2); (d) 3.2 μM HdrA2 (6.2 nmol of F_420_ reduced/min/mg of HdrA2). (B) Kinetic parameters for Fd:F_420_ oxidoreductase activity of HdrA2. The reaction mixtures (0.5 ml) contained 0.1 mg CODH/ACS, 2.2 μM Fdx, 2.1 μM HdrA2, and different amounts of F_420 _in 50 mM MOPS buffer (pH 7.0). 1 mU indicates that 1 nmol of F_420_ is reduced per minute. (Inset) Double-reciprocal plot. Download FIG S5, DOCX file, 0.1 MB. Copyright © 2017 Yan et al. 2017 Yan et al. This content is distributed under the terms of the Creative Commons Attribution 4.0 International license.

**FIG 3:**
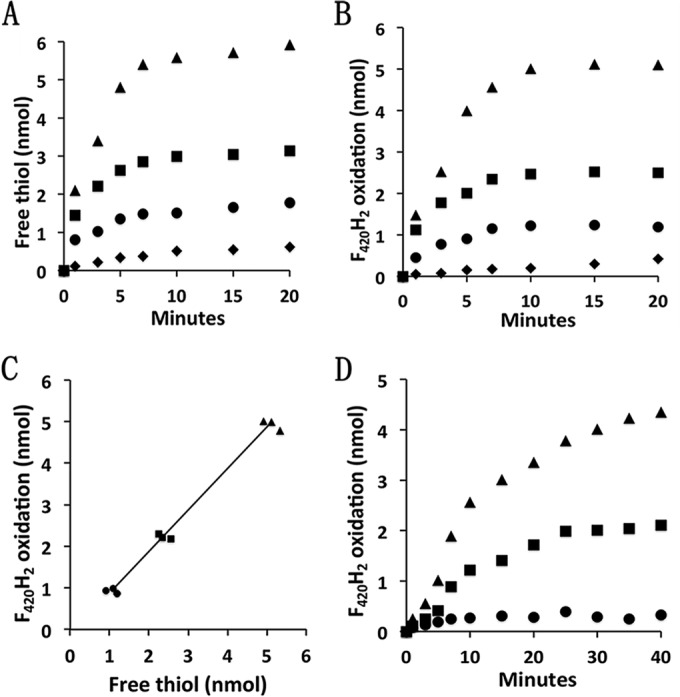
Electron bifurcation of electron pairs from F_420_H_2_ to Fdx and CoMS-SCoB. The reaction mixtures (0.5 ml) contained 1.6 μM HdrA2, 1.8 μM HdrB2C2, 12.5 μM F_420_H_2_, and the indicated amounts of CoMS-SCoB or Fdx in 50 mM MOPS buffer (pH 7.0). The atmosphere was 100% N_2_, and the temperature was 21°C. Reactions were initiated by the addition of HdrA2 or HdrB2C2. (A) Time course for Fdx-dependent production of free thiols in the presence of 50 μM CoMS-SCoB. Symbols: ▲, 5.2 nmol Fdx; ■, 2.6 nmol Fdx; •, 1.3 nmol Fdx; ⧫, no Fdx. (B) Time course for Fdx-dependent oxidation of F_420_H_2_ in the presence of 50 μM CoMS-SCoB. The symbols are the same as those used for panel A. (C) Amounts of free thiol produced versus the amounts of F_420_H_2_ oxidized in the presence of 1.3, 2.6, and 5.2 nmol Fdx. The amounts in the absence of Fdx were subtracted as a blank control. The results from three replicate experiments are shown. Symbols: ▲, 5.2 nmol Fdx; ■, 2.6 nmol Fdx; •, 1.3 nmol Fdx. (D) CoMS-SCoB-dependent F_420_H_2_ oxidation in the presence of 5.2 nmol Fdx. Symbols: ▲, 2.4 nmol CoMS-SCoB; ■, 1.2 nmol CoMS-SCoB; •, no CoMS-SCoB added.

The ability of HdrA2 to interact with F_420_ prompted asking whether HdrA2 together with HdrB2C2 is able to bifurcate electron pairs from F_420_H_2_ for reduction of Fdx and CoMS-SCoB. Figure 3A and B are representative of results showing a dependence on Fdx for free thiol (HSCoM and HSCoB) formation and F_420_H_2_ oxidation in the presence of CoMS-SCoB. A replot (Fig. 3C) of the replicated data shows one free thiol produced from CoMS-SCoB for each F_420_H_2_ oxidized, a result consistent with simultaneous reduction of Fdx. These results show that HdrA2B2C2 catalyzes a thermodynamically favorable (Δ*G*°′ =−38.6 kJ), flavin-based, coupling of the endergonic one-electron reduction of Fdx (*E*_0_′ of ca. −520 mV) with F_420_H_2_ (*E*_0_′ of −380 mV) to the exergonic reduction of CoMS-SCoB (*E*_0_′ of −140 mV) (Table S2) as previously predicted (29). Considering the inability of HdrA2B2C2 to utilize Fdx^1−^ as an electron donor (Fig. 2B), the results indicate the bifurcation proceeds according to equation 6. Figure 3D shows the dependence of F_420_H_2_ oxidation on two limiting concentrations of CoMS-SCoB and a fixed nonlimiting concentration of Fdx that is stoichiometrically consistent with equation 6.

(6) $$2{\text{F}}_{420}{\text{H}}_{2}+2\text{Fdx}+\text{CoMs-SCoB}\to 2{\text{FDx}}^{1-}\text{+ HSCoM}+\text{HSCoB}+2{\text{F}}_{\text{420}}$$

### Fdx:CoMS-SCoB oxidoreductase activity in cytoplasmic and membrane fractions of acetate-grown *M. acetivorans*.

During growth of *M. acetivorans* with acetate, the reduced Fdx generated by oxidation of the carbonyl group donates its electrons to a membrane-bound electron transfer chain culminating with membrane-bound HdrDE that reduces CoMS-SCoB to the corresponding sulfhydryl forms of the cofactors (equation 2). The membrane-bound electron transport is coupled to H^+^ and Na^+^ translocation, forming gradients that drive ATP synthesis (30). Acetate-grown cells also upregulate genes encoding a homolog of HdrABC from CO_2_-reducing methanogens (HdrA1B1C1), HdrA2 and HdrB2C2 (18, 19, 31). Thus, it is hypothesized that either HdrA1B1C1 or HdrA2B2C2 catalyze cytoplasmic Fdx:CoMS-SCoB oxidoreductase activity in acetate-grown cells (19, 25, 31) for which biochemical evidence of cytoplasmic oxidoreductase activity is shown in Table 2. Of the total heterodisulfide reductase activity present in the extracts, a corresponding amount, namely, 56 and 29%, was recovered in the cytoplasmic and membrane fractions, respectively. The cytoplasmic fraction contained approximately 50% of the total oxidoreductase activity in extracts. Less than 10% of the total activity was recovered in the membrane fraction with the location of the remaining 40% unexplained. Nonetheless, the results support a cytoplasmic Fdx:CoMS-SCoB oxidoreductase system that accounts for approximately half of the total activity in acetate-grown cells.

**TABLE 2:** Fdx:heterodisulfide oxidoreductase and heterodisulfide reductase activities in acetate-grown *Methanosarcina acetivorans*

Fraction	Fdx:heterodisulfide oxidoreductase activity^a^				Heterodisulfide reductase activity^b^	
	CO/CODH-dependent		NADPH/FNR-dependent		Total activity (U)	Sp act (U/mg)
	Total activity (mU)	Sp act (mU/mg)	Total activity (mU)	Sp act (mU/mg)		
Extract^c^	2,839 ± 300	17.0 ± 1.8	2,956 ± 301	17.7 ± 1.8	119 ± 12.8	0.73 ± 0.08
Cytoplasmic	1,579 ± 75	10.6 ± 0.5	1,401 ± 164	9.4 ± 1.1	66.5 ± 5.9	0.44 ± 0.04
Membrane	193 ± 2	8.4 ± 0.1	223 ± 16	9.7 ± 0.7	34.4 ± 0.6	1.48 ± 0.03

^a^ One unit is defined as micromoles of sulfhydryl produced per minute.

^b^ One unit is defined as micromoles of reduced methyl viologen oxidized per minute.

^c^ A sample from the same extract was loaded onto the sucrose gradient for preparation of cytoplasmic and membrane fractions.

## DISCUSSION

The results have produced several milestones that provide a greater comprehensive understanding of the HdrABC class. Fusion of the MvhD homolog to the HdrA domain of HdrA2 is an unusual feature that, together with unusual catalytic capabilities reported here, identifies HdrA2B2C2 as representative of a previously unrecognized HdrABC subclass distributed in diverse species from the domains *Bacteria* and *Archaea*.

### Intersubunit electron transfer.

**FIG 4:**
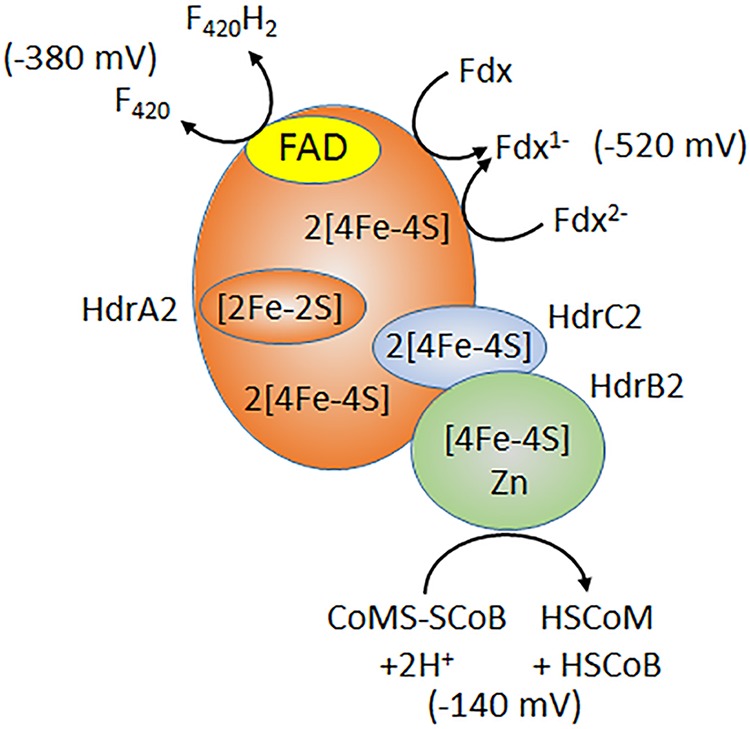
Electron transfer reactions catalyzed by HdrA2B2C2. The redox potentials are standard potentials at pH 7.0 described elsewhere (29). The value for Fdx from *M. acetivorans* is assumed to be similar to estimates (29, 61).

The individual expression and characterization of recombinant HdrA2, HdrB2, and HdrB2C2 provided an experimental approach to identify roles for each subunit previously unknown for any HdrABC homolog (13). The results show that the flavin-containing HdrA2 interacts with F_420_ and Fdx and that HdrA2B2C2 bifurcates electron pairs from F_420_H_2_ directed to the reduction of Fdx and CoMS-SCoB. It was also shown that, although HdrC2 has no role in catalysis, this subunit mediates electron transfer from HdrA2 to the catalytic HdrB2. These results establish the path of electron transfer from Fdx to CoMS-SCoB (Fig. 4) that in all probability generally applies to the HdrABC class.

### Role for the Fdx:CoMS-SCoB oxidoreductase activity of HdrAB2C2 in the pathway for conversion of acetate to CH_4_.

**FIG 5:**
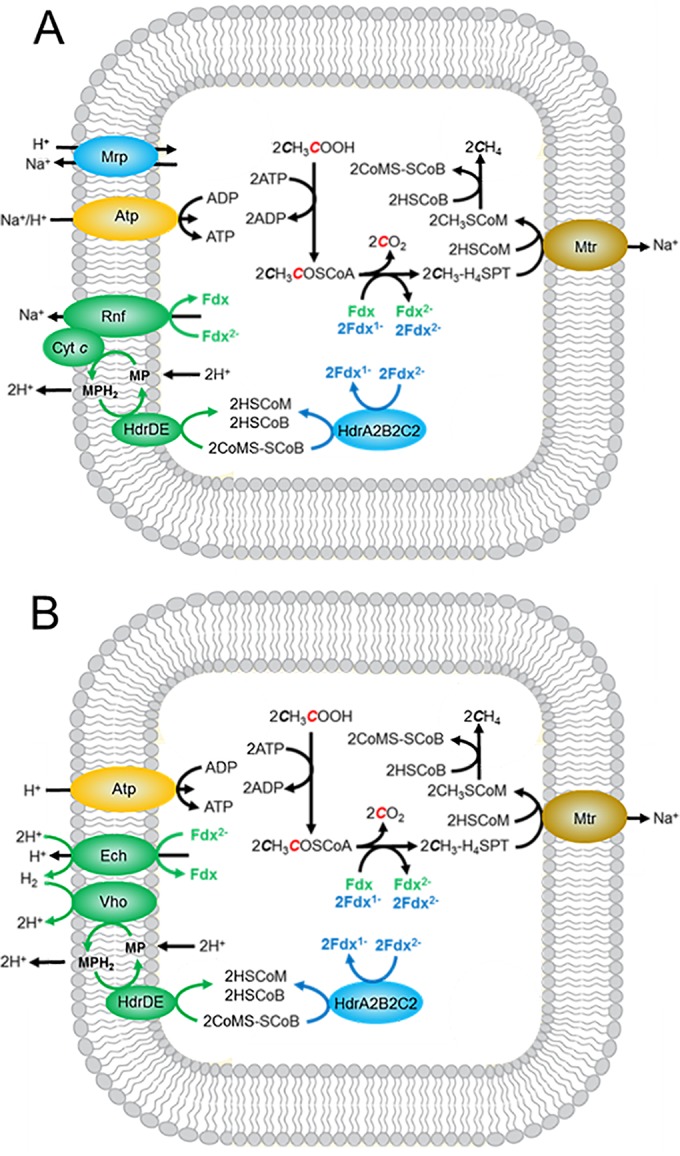
Pathways of acetate conversion to methane by *Methanosarcina* species showing electron transport chains diverging from reduced ferredoxin. (A) Pathway not requiring H_2_. (B) Pathway requiring H_2_. Ech, proton-pumping H_2_-evolving hydrogenase; Vho, H_2_ uptake hydrogenase; Mrp, multisubunit Na^+^/H^+^ antiporter; Atp, ATP synthase; Rnf, Rnf complex; Cyt *c*, multiheme cytochrome *c*; Fdx, ferredoxin; H_4_SPT, tetrahydrosarcinapterin; MP, methanophenazine; HdrDE, membrane-bound heterodisulfide reductase; HdrA2B2C2, cytoplasmic heterodisulfide reductase; HSCoM, coenzyme M; HSCoB, coenzyme B; Mtr, methyltransferase.

Coenzyme F_420_ does not participate in the pathway of acetate conversion to methane, ruling out a role for the F_420_H_2_ bifurcating activity of HdrA2B2C2. However, a role is envisioned for the Fdx:CoMS-SCoB oxidoreductase activity of HdrAB2C2 as shown in Fig. 5. The canonical HdrABC class is a cytoplasmic enzyme that contains flavin, whereas the HdrDE class is membrane bound, flavin free, and contains heme (32, 33). The cytoplasmic HdrABC class is considered specific to obligatory CO_2_-reducing methanogens, and the membrane-bound HdrDE class is considered specific to pathways converting acetate and methylotrophic substrates (methanol, methylamines, and methylsulfide) to CH_4_. However, homologs of the cytoplasmic HdrABC class are also encoded in the genomes of acetotrophic and methylotrophic species from the order *Methanosarcinales* consistent with an auxiliary function. Our results indicate HdrA2B2C2 is responsible for cytoplasmic Fdx:CoMS-SCoB oxidoreductase activity of *M. acetivorans* when metabolizing acetate to CH_4_. This conclusion is further supported by the reported upregulation of *hdrA2*, *hdrB2*, and *hdrC2* in response to growth with acetate and genetic analyses indicating a role for HdrA2 while excluding a role for the canonical HdrA1B1C1 (19, 31, 34). Although HdrB2C2 was shown to catalyze the transfer of electrons from Fdx to CoMS-SCoB, the HdrA2B2C2 complex was shown to be more efficient, which suggests a greater role in cytoplasmic electron transport during conversion of acetate to CH_4_. Genes encoding HdrA2B2C2 of *M. acetivorans* constitute a clade with homologs of other acetotrophic methanogens, further supporting a role in the pathway of acetate conversion to methane (19).

The *hdrA2* gene in *M. acetivorans* is in an operon with a gene encoding a putative polyferredoxin that is upregulated during acetotrophic growth, which suggests a potential role in mediating electron transfer to HdrA2B2C2 (18, 19, 31). Attempts to produce the recombinant protein in *E. coli* strain BL21(DE3) Δ*iscR* resulted in preparations that contained only a partial complement of Fe-S clusters that resisted reconstitution. The preparations were unable to replace Fdx or stimulate reduction of CoMS-SCoB in complete reaction mixtures. Therefore, no definitive conclusions can be drawn regarding the role of the polyferredoxin except that it is not necessary for the transfer of electrons from CODH/ACS to HdrA2B2C2.

A few *Methanosarcina* species contain a hydrogenase (Ech) that oxidizes the Fdx with production of H_2_ and translocation of H^+^ (24). A second hydrogenase (Vho) oxidizes H_2_ and reduces methanophenazine (MP), a quinone-like electron carrier that donates electrons to HdrDE and translocates an additional 4H^+^. Thus, together with the membrane-bound methyltransferase (Mtr), four coupling sites produce Na^+^ and H^+^ gradients that drive ATP synthesis. Most *Methanosarcina* species, represented by *M. acetivorans*, do not contain functional hydrogenases. Instead, *M. acetivorans* contains the membrane-bound Rnf complex that accepts electrons from Fdx and reduces MP mediated by cytochrome *c* (25). The membrane-bound transfer of electrons from Fdx to CoMS-SCoB supports Na^+^ translocation by the Rnf complex and H^+^ translocation by HdrDE (35). Thus, a possible three coupling sites generate Na^+^ and H^+^ gradients that together drive ATP synthesis in *M. acetivorans* (30). The free energy available from conversion of acetate to CH_4_ and CO_2_ under standard conditions of equimolar reactants and products (Δ*G*°′ = −36 kJ) provides only a marginal amount of energy for growth considering the ATP requirement for activating acetate to acetyl-CoA in the first step in the pathway (Δ*G*°′ = +31.8 kJ) (36). Thus, growth with acetate is at the extreme thermodynamic limit, requiring extraordinary mechanisms for maximizing the thermodynamic efficiency. Our finding of a cytoplasmic Fdx:CoMS-SCoB oxidoreductase system in acetate-grown *M. acetivorans*, and reconstitution of an active HdrA2B2C2, supports the divergent electron transport pathways from Fdx to CoMS-SCoB shown in Fig. 5A. *Methanosarcina mazei* and *Methanosarcina barkeri*, species that produce and consume H_2_ during growth on acetate, also encode HdrA2, HdrB2, and HdrC2 homologs with greater than 80% amino acid sequence identity, predicting these species also synthesize HdrA2B2C2 participating in a soluble electron transport pathway (Fig. 5B). Acetyl-CoA-dependent methanogenesis catalyzed by *M. barkeri* cell lysate is not dependent on the membrane fraction consistent with a cytoplasmic electron transport system (37). For *Methanosarcina* species with documented respiratory control (38), HdrA2B2C2 provides a mechanism for modulating cytoplasmic versus membrane-bound electron transport proportional to ATP demand. We posit that the HdrA2B2C2-dependent electron transport allows cells to maximize thermodynamic efficiency by circumventing membrane-bound electron transport when nonstandard concentrations of acetate encountered in the environment constrain the free energy available for multiple ion translocation sites. With this mechanism, *Methanosarcina* species could maximize the thermodynamic efficiency and metabolize acetate more rapidly, thereby outcompeting others for acetate. Indeed, *M. acetivorans* must compete with the more thermodynamically favorable acetate utilization by sulfate-reducing species (CH_3_COO^−^ + SO_4_^2−^ → 2HCO_3_^−^ + HS^−^; Δ*G*°′ = −71.7 kJ) in the marine environment where *M. acetivorans* was isolated (17, 39).

### A proposed role for the F_420_H_2_ bifurcation activity of HdrA2B2C2 homologs in Fe(III)-dependent anaerobic CH_4_ oxidation (ANME).

**FIG 6:**
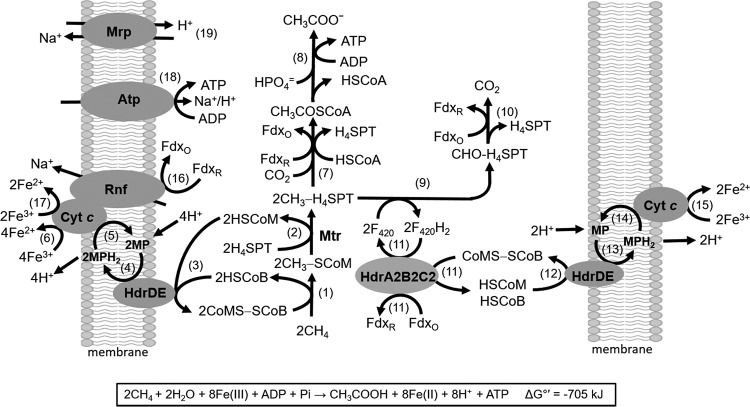
Pathway proposed for anaerobic CH_4_ oxidation (ANME) by *M. acetivorans*. The reaction numbers are shown in parentheses in the figure. CoA-SH, coenzyme A; H_4_SPT, tetrahydrosarcinapterin; Fdx_R_, reduced ferredoxin; Fdx_O_, oxidized ferredoxin; HSCoM, coenzyme M; HSCoB, coenzyme B; MP, methanophenazine; HdrDE, membrane-bound heterodisulfide reductase; HdrA2B2C2, cytoplasmic heterodisulfide reductase; Rnf, Rnf complex; Cyt *c*, cytochrome *c*; Atp, ATP synthase; Mrp, sodium/proton antiporter; Mtr, membrane-bound methyltransferase. Enzymes and thermodynamic calculations for each reaction are shown in Table S2.

Although the F_420_H_2_ bifurcation activity of HdrA2B2C2 is unanticipated in the pathway of acetate conversion to methane, a role for this activity can be envisioned for homologs in pathways of Fe(III)-dependent anaerobic methane oxidation as shown in Fig. 6. For more than a decade, it was assumed that ANME required a consortium of at least two metabolic groups. However, recent reports indicate uncultured *Archaea* species in ANME group 2 (ANME-2) environments oxidize CH_4_ alone, albeit dependent on Fe(III) as a direct electron acceptor (40, 41). Furthermore, genomic analyses of an ANME-2a environment implicate a role for HdrABC homologs and acetate as a product (9). Homologs of HdrA2 and HdrB2 are encoded in the genome of “*Candidatus* Methanoperedens nitroreducens” (see Fig. S1 in the supplemental material) consistent with roles in the NO_3_^−^-dependent ANME pathway of this and possibly other ANME species dependent on external electron acceptors (42). Notably, Fe(III)-dependent oxidation of CH_4_ was recently reported for an enrichment culture containing archaea of the order *Methanosarcinales* related to “*Candidatus* Methanoperedens nitroreducens” (43). Isolated ANME species have not been reported; however, *M. acetivorans* is capable of Fe(III)-dependent conversion of CH_4_ to acetate and is phylogenetically related to uncultured species identified in ANME-2 consortia (44). Our current understanding leads to a proposed ANME pathway for *M. acetivorans* involving HdrA2B2C2 (Fig. 6; Table S2) that may also be operable in ANME-2 environments.

The core of the proposed ANME pathway (reactions 1 to 5, 7, 8, 16, 18, and 19) is a reversal of the established acetate-utilizing CH_4_-producing pathway in *M. acetivorans* (45). Table S2 lists thermodynamic calculations for reactions shown in Fig. 6. Reactions 1 and 2 in the ANME pathway are thermodynamically unfavorable (Δ*G*°′ = +121 kJ), requiring oxidation of HSCoM and HSCoB coupled to reduction of Fe(III) (reactions 1 to 6), yielding an overall Δ*G*°′ of −176 kJ (Table S2). The oxidation is catalyzed by HdrDE with transfer of electrons to methanophenazine (MP) and cytochrome *c* where Fe(III) is the terminal electron acceptor, thereby pulling reactions 1 and 2. Scalar proton translocation is accomplished by a “Q loop” mechanism involving MP and driven by reactions 3 to 6 (Δ*G*°′ = −297 kJ). Acetate-grown *M. acetivorans* is rich in multiheme cytochrome *c* that shuttles electrons between the Rnf complex and MP (25, 46). Reduction of Fe(III) at cytochrome *c* of *M. acetivorans* is consistent with the documented role of multiheme *c*-type cytochromes as electron shuttles to Fe(III) minerals outside microbial cells (47, 48). Moreover, metagenomic analyses of ANME environments reveal homologs of genes encoding *c*-type cytochromes and Rnf complexes hypothesized to function in ANME pathways (9, 41, 49).

The product of reaction 2 (CH_3_-H_4_SPT) is metabolized by divergent pathways leading to either acetate or CO_2_. The acetogenic pathway begins with reaction 7 (Δ*G*°′ = −40.5 kJ) requiring reduced Fdx for reduction of CO_2_ that provides the carbonyl group of acetyl-CoA. The requisite reduced Fdx is generated in the pathway oxidizing the methyl group of CH_3_-H_4_SPT to CO_2_ (reactions 9 to 15). The oxidation pathway is identical to that which functions in the dismutation of methylotrophic substrates to CO_2_ and CH_4_ wherein reaction 9 involves multiple steps, including two oxidations dependent on F_420_ as the electron acceptor (50). The F_420_H_2_ is oxidized by HdrA2B2C2 with bifurcation of electrons directed to Fdx and CoMS-SCoB (reaction 11). The bifurcation (Δ*G*°′ = −38.6 kJ) is coupled to reactions 12 to 15 (Δ*G*°′ = −148.5 kJ), reducing Fe(III) with an overall Δ*G*°′ of −187.1 kJ. Reactions 12 to 15 are catalyzed by the same proteins and electron carriers as those used in reactions 3 to 6 albeit with different stoichiometry. Scalar proton translocation generates a proton gradient (high outside) accomplished by a “Q loop” mechanism involving MP. The exergonic oxidation of Fdx and reduction of Fe(III) in reactions 16 and 17 (Δ*G*°′ = −249.3 kJ) drives a vectorial translocation of Na^+^ consistent with the previously reported reduction of cytochrome *c* and pumping of Na^+^ by Rnf (25, 35). The thermodynamically unfavorable reaction 2 (Δ*G*°′ = +29.2 kJ) is catalyzed by the membrane-bound methyltransferase (Mtr) and driven by the Na^+^ gradient. ATP synthesis is catalyzed by the ATP synthase (reaction 18) dependent on the Na^+^ and H^+^ gradients (30). ATP is also synthesized by substrate level phosphorylation (reaction 8) catalyzed by phosphotransacetylase and acetate kinase. As previously proposed, multisubunit Na^+^/H^+^ antiporter (Mrp) functions to adjust the ratio of Na^+^/H^+^ (reaction 19) optimal for the ATP synthase and methyltransferase (51, 52). Finally, the stoichiometry shown in Fig. 6 assumes a low availability of Fe^3+^ that limits the Fe(III)-dependent oxidation of Fdx (reactions 16 and 17), allowing for the Fdx-dependent synthesis of acetate (reactions 7 and 8). Reactions 16 and 17 are more thermodynamically favorable than reactions 7 and 8 (Table S2); therefore, complete oxidation of CH_4_ to CO_2_ would be expected when Fe^3+^ is nonlimiting. Thus, the availability of Fe^3+^ in the native environment is expected to modulate the stoichiometry of CH_4_ oxidation to acetate and CO_2_. It is also possible that reactions 7 and 10, or reactions 7 and 11, are spatially coupled, circumventing the Fe(III)-dependent oxidation of reduced Fdx (Fdx_R_) by reactions 16 and 17, resulting in the stoichiometry shown in Fig. 6 when Fe(III) is nonlimiting.

Additional electron bifurcating roles are possible for HdrA2B2C2 homologs in nonmethanogenic species with diverse metabolisms (Fig. S1) that may replace F_420_H_2_ (*E*_0_′ = −380 mV) with the analogous two-electron carrier NADH (*E*_0_′ = −320 mV) and replace CoMS-SCoB with disulfides such as the DsrC protein postulated for the FlxlABCD-HdrABC bifurcating complex of *Desulfovibrio vulgaris* (12).

### Conclusions.

HdrA2B2C2 represents a subclass of the HdrABC class with homologs in diverse species of the domains *Bacteria* and *Archaea*. Results revealed a previously unknown electron bifurcation system and intersubunit electron transport pathway generally applicable to the HdrABC class. Properties of HdrA2B2C2 predict that homologs participate in anaerobic CH_4_ oxidation pathways and that HdrA2B2C2 is essential for optimal growth of acetotrophic methanogens in native environments.

## MATERIALS AND METHODS

### Cell growth and materials.

Acetate-grown *Methanosarcina acetivorans* was mass cultured and harvested as previously described (53). *E. coli* strain BL21(DE3) Δ*iscR* was a gift from J. Golbeck. HSCoB and CoMS-SCoB was a gift from T. Wood. All chromatography columns, resins, and prepacked columns were purchased from GE Healthcare. Purification of F_420_ from methanol-grown *M. acetivorans* cells and preparation of F_420_H_2_ was performed as described elsewhere (54). All other chemicals were purchased from Sigma-Aldrich or VWR International.

### Cloning of *M. acetivorans* genes.

Genes encoding Fdx (MA0431), HdrA2 (MA2868), and HdrB2 (MA4237) were amplified by PCR from genomic DNA using primers shown in Table S1  in the supplemental material and cloned into pET22b (Novagen) using In-Fusion (Clontech) for expression of the proteins with a C-terminal His_6_ tag. Genes encoding HdrC2 (MA4236) and HdrB2 (MA4237) were amplified by PCR and cloned into the first and second site, respectively, of pETDuet (Novagen) for coexpression with a C-terminal His_6_ tag on HdrB2. All constructs were validated by sequencing.

10.1128/mBio.02285-16.7 TABLE S1  Primers used for amplification of genes and construction of plasmids. Download TABLE S1, DOCX file, 0.01 MB. Copyright © 2017 Yan et al. 2017 Yan et al. This content is distributed under the terms of the Creative Commons Attribution 4.0 International license.

10.1128/mBio.02285-16.8 TABLE S2  Standard Gibbs free energy values for reactions in the Fe(III)-dependent ANME pathway proposed for *Methanosarcina acetivorans*. Download TABLE S2, DOCX file, 0.02 MB. Copyright © 2017 Yan et al. 2017 Yan et al. This content is distributed under the terms of the Creative Commons Attribution 4.0 International license.

10.1128/mBio.02285-16.9 TABLE S3  Gibbs free energy of metabolite formation (Δ_f_G^t^) calculated at 25°C, pH 7, and ionic concentration of 0.25 M. Download TABLE S3, DOCX file, 0.03 MB. Copyright © 2017 Yan et al. 2017 Yan et al. This content is distributed under the terms of the Creative Commons Attribution 4.0 International license.

### Expression and purification of recombinant proteins.

The expression plasmids (Table S1) were transformed into *E. coli* strain BL21(DE3) Δ*iscR* and grown with 100 µg/ml ampicillin. A single colony was used to inoculate 100 ml of LB medium buffered with 50 mM morpholinepropanesulfonic acid (MOPS) (pH 7.4) and supplemented with 100 μg/ml of ampicillin that was incubated at 37°C for 12 h. The starter culture was subcultured (1:50 dilution) in 3 liters of the above medium supplemented with 1 mM ferric ammonium citrate that was contained in a spinner flask (Chemglass) modified to allow sparging with 100% Ar. Cultures were grown aerobically at 37°C to an optical density at 600 nm (OD_600_) of 0.6 that was then supplemented with 1 mM cysteine and induced with 200 µM isopropyl-β-d-thiogalactopyranoside (IPTG). Anaerobic metabolism was facilitated by the addition of glucose (0.5%, wt/vol) and sodium fumarate (25 mM) followed by incubation at 21°C for 20 h with sparging. Cells were harvested by centrifugation in sealed tubes containing 95% N_2_–5% H_2_, resuspended (1:5, wt/vol) in 50 mM Tris (pH 8.0) containing 10% glycerol (buffer C), and stored at −80°C until use. The anaerobic purification of recombinant proteins is described in Text S1. Purified HdrA2 was reconstituted with FAD by anaerobic incubation at 4°C for 10 h in buffer containing 0.1 mM FAD and passed through a PD-10 column to remove excess flavin.

10.1128/mBio.02285-16.1 TEXT S1  Supplemental Materials and Methods. Download TEXT S1, DOCX file, 0.02 MB. Copyright © 2017 Yan et al. 2017 Yan et al. This content is distributed under the terms of the Creative Commons Attribution 4.0 International license.

### Purification of CODH/ACS.

The five-subunit enzyme was purified from acetate-grown *M. acetivorans* as described previously with modifications noted in Text S1 (55). A typical preparation reduced the recombinant Fdx (33.3 μM) at a rate of 55.3 nmol/min/mg CODH/ACS.

### Enzyme assays.

All enzyme assays were performed at 21°C in stoppered cuvettes containing the indicated atmosphere. Heterodisulfide reductase forward and reverse activities were performed as described elsewhere with modifications noted in Text S1 (56). Fdx:CoMS-SCoB oxidoreductase activity was performed as described in Text S1.

### Other analytical procedures.

All protein concentrations, except for Fdx, were determined with either the Bradford assay kit (Bio-Rad Laboratories) or Pierce assay kit (Thermo Scientific) that gave similar results. The protein concentration for Fdx was determined using the ε_390_ of 30 mM^−1^ cm^−1^ (57). Iron and acid-labile sulfur contents were determined as described elsewhere (58, 59). The flavin content of HdrA2 was determined by UV-Vis and fluorescence spectrometry as described elsewhere (60). For the electron bifurcation experiments, F_420_H_2_ oxidation was determined by monitoring the fluorescence intensity with excitation at 420 and emission at 480 nm.
