# Supplementary material for: A Ferredoxin- and F420H2-Dependent, Electron-Bifurcating, Heterodisulfide Reductase with Homologs in the Domains Bacteria and Archaea
Source: mBio. 2017 Feb 7;8(1):e02285-16. doi: 10.1128/mBio.02285-16 (PMC5296606; doi:10.1128/mBio.02285-16)
Supplement: FIG S1 [file mbo001173173sf1.docx]

**Figure S1.**

*** * * ***

WP_011022820 1 MRIGVYICHCGLNIAGVIDVSALEAMAN-ELEDVVLAREVQFLCSDSGQEGIIKDIKDNKLDRVVVAACSPRLHEK 75

WP_048037482 1 MRIGVYICHCGLNIAGVIDVSALEEMAG-ELEDVVLAREVQFLCSDSGQEGIIKDIKDNKIDRVVIAACSPRLHEK 75

WP_011307542 1 MRIGVYICHCGLNIAGVIDVLALQEMAA-KLEDVVLAREVQFLCSDSGQEGIIKDIKENKIDRVVIAACSPRLHEK 75

WP_023843920 1 MRIGVYICHCGLNIAHTINVKSLRDKVS-ELNDVALVKDIQFMCSDSGQDSIVQDITDLDLNHILVAACSPHLHEQ 75

WP_015324480 1 MRIGVYICHCGLNIAHVINVHSLQEKVS-KLKDVVLVKDLQFMCSDSGQDSIAEDIKAHDLDHILVAACSPKLHEP 75

WP_048194718 1 MRIGVYICHCGLNIASVINMDALHQKVE-EMEDVALVKDIQFMCSDFGQEQLIDDIKENNIDRILVAACTPKLHEP 75

WP_013897960 1 MKIGVYICHCGLNIANVINVDILHSKVE-KLEDVAVVRDVQFMCSDAGQEYIIEDIGDNNLDRILVAACSPKLHEQ 75

WP_013194860 1 MKIGIYICHCGLNIAHVINMDTLQDKIKkEFKDIEIVRDIQFMCSDTGQEAIIEDIKEHNIDRVLIAACSPKLHEK 76

WP_013037418 1 MRIGVYICHCGLNIAGVVDIETLLKQVE-SLDDVVVASDIQFMCSDSGQEKITKDIEEHDVERVLVAACTPKLHET 75

WP_048089472 1 MPTGVYICHCGSNIAGTIDVEDVREHAR-MLKGVAVARDIPFACGDSGQEQIKKDIQEFRLDRIVMAACSPRLHEV 75

WP_014586170 1 MRIGVYICHCGLNIAGVLEPSALAEFAK-TLPGVEVARDLQFTCSDTGQDMIKEDIAEHKLESIVVAACSPRLHEP 75

WP_042684393 1 MRIGVFVCHCGQNIAGALDTSRVLECAL-SEEGVAHAEELQFACSQEGQRAIQSAIVEHKLDRIVVAACSPHLHEP 75

WP_012964674 1 MRIGVFVCHCGLNIARIVDVQEVVNYAK-NLEDVYFSTDLKYACSDSGQEEIIKAIKENRLDAVVIAACSPKLHEH 75

WP_010878733 1 MRIGVFVCHCGLNIARVVDVKELVEYAK-TIDGVVHAEDIDYACSDSGQEEILNAIKEKNLDAFVVAACSPKLHEP 75

KPL16356 1 [ 8]LRIGVYVCHCGLNIAGSVDCEAVTEYAR-GLDDVVVASDNKYTCSDPGQDIIKNEIREHKLNRVVVASCTPRLHEP 83

KPV63986 1 [17]IRIGVYVCHCGLNIAGSVDCEEVARFAA-TLPHVVLSKDNRYMCSDPGQELLKRDIREQKLSRVVVASCSPRLHEP 92

WP_051309217 1 [ 8]LRIGVYVCHCGLNIAGVVDCGAVSEYAA-TLPDVVLSRHNAYTCSEPGQNQIKQDIADHKLNRVVVASCSPRLHEP 83

WP_041286569 1 [ 4]LRIGVYVCHCGKNIGGTVRCDEVAEYAS-HLPGVILAKDSLYTCSEPGQEQIKHDIREHDLNRVVVASCTPRLHEP 79

KPJ88130 1 [13]PRIGVYVCHCGLNIGGSLDCEAVAMHAM-NLDNVVYTEHQLYTCSEPGQEKIKKDIAEHNLNRIVVAACSPRMHEP 88

#

WP_011022820 76 TFRHVMEKAGLNPYLMEMVNIREQCSWVHADDPQMATQKAFDLIRMGVAKARFLRELSATNSKASRNVLIIGGGVAGIEA 155

WP_048037482 76 TFRHVMEKADLNPYLMEMVNIREQCSWVHADDPQMATQKAFDLIRMGVAKARFLRELSATSSKASRNVLIIGGGVAGIEA 155

WP_011307542 76 TFRHVMEKAGLNPYLMEMVNIREQCSWVHADDPQMATQKAFDLIRMGVAKAKFLKELSATSSKASRNVLIIGGGVAGIEA 155

WP_023843920 76 TFKRVLEKAGLNPFMLEMVNIREQCSWVHMDNPQMATQKAFDLIRMGIARLKLLDPLQLKKVPAKKDVMVIGGGVAGIEA 155

WP_015324480 76 TFRRVLQKAGLNKYMLEMVNIREQCSWVHMAEPQLATQKAFDLIRMGLARLKLLDPLQIKTVTANKSVLIIGGGVAGIEA 155

WP_048194718 76 TFKRVLEKAGINPYLLEIANIREQCSWVHMHNHSMATQKAFDLIKMGVAKLKLLAPLQIRTFKANKDVLVIGGGVAGIEA 155

WP_013897960 76 TFRRVIEKAGINPYLLEMVNIREQCSWVHGNNPQMATQKAFDLIKMGLARLKHSNPLDIEKFRINTDVLVIGGGVTGIEA 155

WP_013194860 77 TFQQVIENAGVNPYLLEIVNIREQCSWVHKQHPEMATQKAFDLIKMGVAKLKNLEPLEVKKVSINRNVLVIGGGVAGIEA 156

WP_013037418 76 TFRNVLEKSGINPFLLEIVNIREQCSWVHMENPRMATQKAFDLIKMGVAKLRFLKPLKIQSTPVSKEVLVIGGGVAGIEA 155

WP_048089472 76 TFRRLLEQSGLNPHLLGMVNIREQGSWVHSDEDGLATQKAKDLVSMGVARVALLTPLDKKTIPANKDVLVIGAGVAGIEA 155

WP_014586170 76 TFRRVISEAGINPFMLEMANIREQCSWVHMDEPPQAQEKAKDLIRMAVAKAALLNPLQGETMPVSREVLVIGGGVAGIQA 155

WP_042684393 76 TFRRAVEGL-INPYLVECTNIREQCSWVHTDRAR-ATRKACDLVRMGIARARHLEPLKPRRIVVNRDVLVIGGGVAGITA 153

WP_012964674 76 TFRKAAMRAGLNPYMVLMANIREQCSWVHQEHPKAATEKAKDLVRMAVAAARKLEPLSRKRIEVKKSAVVIGGGVAGIEA 155

WP_010878733 76 TFRRVAIRAGLNPYMVEIANIREQCSWVHQAKPKAALAKAKDLIRMAVAKARTNRPLERRKAEIERSVAVIGGGVAGIEA 155

KPL16356 84 TFRKACEDAGLNPYLFEMANIRDQCSWVHLYDREGATQKAKDLVKMAVARARLLTPQYETEVPVTRKALVIGGGVAGIQA 163

KPV63986 93 TFRKACEEAGLNRYLFEMANIREQCSWVHLYEREMATEKAKDLVKMAVAKAAFLEPAEESEVPIIKKALVIGGGVAGIQA 172

WP_051309217 84 TFRQCVAEAGLNPYLLDMANLREHCSWVHCNDKQGATQKACDLVRASVARTRLLQECHEAQIAVTPTTLVVGGGVAGIQA 163

WP_041286569 80 TFRAACESAGLNPYLLEMANIREHCSWVHLHDKDAATEKAKDLVAMAVNRAARLKPQIEVTVPVTRKAMVIGGGVAGIQA 159

KPJ88130 89 TFRSCIKAAGLNQYLLEMANIREHCSWVHLHDREAATEKAKDLVRMSVARARLLEPQEEIDIPVIQKALVIGGGVAGIQT 168

*** ***

WP_011022820 156 ALNLAEAGFPVTMVEKESTIGGKMALMNEVFPTNDCSICVLAPKMTEVQNHPNITLYTYSEVTDISGSVGKFHVRVKRKP 235

WP_048037482 156 ALNLAEAGFPVTMVERESTIGGKMALMNEVFPTNDCSICVLAPKMTEVQNHPNITLYTYSEVTDISGSVGKFHVRVTRKP 235

WP_011307542 156 ALNLAEAGFPVTMVEKESTIGGKMALMNEVFPTNDCSICVLAPKMTEVQNHPNITLYTYSEVTDISGSVGKFHVKVTRKP 235

WP_023843920 156 ALTLANSGHHVIMVEKEPTIGGKMALLNEVFPTNDCSICVLAPKMTDVNQHPNIDLITLAEVTDVSGPVGNFNVTVTRQP 235

WP_015324480 156 ALTLADSGYHVHMVEKEPTIGGKMALLNEVFPTNDCSICVLAPKMTDVQQHPSVDLMTMAEVSEVRGFAGNFQVTVTKKP 235

WP_048194718 156 ALTLADAGTHVYMVEKEPTIGGKMALLNEVFPTNDCSICVLAPKMTDVQNHPNIEMRTYSEITDISGSVGNFNVKGVEHP 235

WP_013897960 156 SLNLADSGYNVHLIEQEPTIGGKMALINEVFPTNDCSICVLAPKMTEVHNHPNITLLTLSQITEITGNVGNFKIKGVKRP 235

WP_013194860 157 ALNLADAGYHVYMVEKEPSIGGKMALLNEVFPTNDCSICVLAPKMTEVSNHPNIELYTNSEITEITGSVGNFTVKGLKKP 236

WP_013037418 156 ALNLADTGYKVHMIEKEPTIGGKMALLNEVFPTNDCSICVLAPKMTEVVNHPDIELHTLSEITAIEGSVGNFEVTGITQP 235

WP_048089472 156 ALHLADMGIKVRLVEKEPTIGGKMALMNEVFPTNDCSLCALAPRMSDVQSHPNIKLYTYSEITKIEGRAGNFKITGVKKP 235

WP_014586170 156 SLDLADCGLSVHLVERRPTIGGYMALLTDVFPTNDCSICVLAPKMTDVYNHPLIDLVTYAEILNIEGSVGRFTVSGVRKA 235

WP_042684393 154 ALELANADFHVHLVERNSSLGGKMALLDKVFPTGDCSICVFAPKMSEAYAHPNITVHTYTEVEDISGHVGSFRIKLRHKP 233

WP_012964674 156 ALTLANAGVKVYLIEKAPTIGGKMATLNEVFPTNDCSICILAPKMSEAFNHENIEVITNAEVLEVSGHVGNFKVKVRKHP 235

WP_010878733 156 ALTLADSGIKVYLIEKNPTIGGHMATLNEVFPTNDCSICILAPKMSDVWNHENIEVITNAEIDEINGSVGNFRIKVIKHP 235

KPL16356 164 ALDLADQDYKVTLVEKEPSIGGKMAQIDKTFPTMDCSICILAPKMSEAGRHPNITLLTNSEVKEVSGYIGNFQIKVLKKA 243

KPV63986 173 ALDLGDTGYKVYLVEKEPSIGGRMAQIDKTFPTMDCSICILAPKMSDVGRHPNIELLTNSEVVEVKGYIGNFRVMVLKKP 252

WP_051309217 164 ALDLANAGTKVILVEKKPSIGGIMAALDKTFPTMDCSICILGPKMTDVGRHPNITLLTLSEVKEIKGFVGNFTVRVLKKA 243

WP_041286569 160 ALDMADAGYKVYLVERTGSIGGRMAQIDKTFPTMDCSICILAPKMSEVGRHPNIELLTLSEIQEVQGHIGNFKAKILKKA 239

KPJ88130 169 ALDLADAGYEVNLVEKEPSIGGRMAQIDKTFPTMDCSICILAPKMAEAGRHPNIRLMTLTEVTSINGYVGNFTVSVMHNA 248

*** * * * * * * ***

WP_011022820 236 RFVLEDKCKGCVDLCSGVCPVEIENPMNYGIGKTRAIYMPIPQSVPQVVLIDPDHCVG CGLCQLACPAEAVDYEQKP 312

WP_048037482 236 RFVLEDKCKGCVDLCSEVCPVEIENPMNYGIGKSRAIYMPIPQSVPQVVLIDPDHCVG CGLCQLACPAEAVDYEQKP 312

WP_011307542 236 RFILEDKCKGCVDLCSAVCPVEIENPMNYGVGKTRAIYMPIPQSVPQVVLIDPDHCVG CGLCLQACPADAVDYEQKP 312

WP_023843920 236 RYVDEDKCKGCVDECGRVCPVEVPNRFDSGLGKTKAINMPIPQAVPQVVYIDNEYCVG CGLCKLACPADAIHYHQKE 312

WP_015324480 236 RYVIEDRCKGCVDECSRVCPVEMANRFDMGMGRTKAINMPIPQAVPQVAYIDSDYCVG CGLCMQACPADAIDYNMKE 312

WP_048194718 236 RYVMIDRCKGCIDQCSNVCPVEIPNPFDSGLGKTKAINMPIPQAVPQSAYIDNEFCVG CGLCKQACPADAIDYNLKE 312

WP_013897960 236 RYVLEDKCKGCVEDCSGVCPVEIPSKFDYGLGKKKAISIPIPQSVPQVAYINSDYCVG CGLCSLACPAEAIDYKQKE 312

WP_013194860 237 RYVSEDKCKGCIEECSSVCPVEISNPFDYGIGKIKAISMPFPQAVPQCAYINDEYCVG CGLCRQVCPADAVDYEQKE 313

WP_013037418 236 RYVSEEKCKGCVDECSRVCPVEIPSRFDSGLGKSRAINIPIPQAVPQVAYIDGDYCVG CGLCAQACPADAVEFEQQT 312

WP_048089472 236 RYVDEKKCKGCIDLCAHVCPIDVPNQFDYGIGARKSIYIPFAQAVPLVACID-EHCVG CGMCRLACPAEAVDFTQRP 311

WP_014586170 236 RFVDEKLCKGCLNECAGVCPVEVPDDYEFGLGKRKAIYMPIPQAVPLVACIDPSACIG CGLCAEACPVDAVKYDQRQ 312

WP_042684393 234 RYI-NEYCKGCIELCSSVCPVEVPNEFDFHLSKRKAIYKPFAQAVPEYAVIDPEACVG CGLCRLACPLDAVDYEQKE 309

WP_012964674 236 RYVDENKCKGCIDDCSSVCPVEVPNEFDYTIGVRKAIYLPIPQSTPLYAAIDWEHCIG CRLCEKACEPKAVDFNQKP 312

WP_010878733 236 RYVDESKCKGCIDDCSSVCPVEIPNEFDYGIGVRKAIYIPIPQSTPLYAAIDWEHCIG CRLCEKACQPKAVDFSQQP 312

KPL16356 244 RFVDEKECTACGD-CADVCPVVRPNEFDVGLATRKAIYTPFAQAVPSAYIINMDDCLG[5]CGKCVDKCEKHAINFDMPD 324

KPV63986 253 RYV-TRDCTACGD-CSKVCPVTAPNEFDVGLATRRAIYTPFAQAVPSTYIIDRNICLN[6]CDKCIKACERRAIDFDMKP 333

WP_051309217 244 RYVHEDMCTACGE-CAKVCPVVLPDEFDQGLSSRRAIYSPFPQAVPSSYLIEMERCLG[5]CGKCVDQCEKQCIDFHMSD 324

WP_041286569 240 RYV-TKECTACGD-CIQACPQLSPDEFNAGLSIRRAIHIPFAQAVPSTFLIDMDRCLN[6]CDRCFQSCSHKCIDFADKD 320

KPJ88130 249 RYV-TSDCSACGD-CSNVCPQFAPNEFDIGLAARKAIYLPFAQAVPSKYLIDMNLCLN[6]CEKCVEACERKCINFNDTR 329

*****

WP_011022820 313 EEIEFEAGAIIVSTGYQLFDASRKKEYGFGKYPDVITNMQLERMLNSAGPTGGRVLVPSTGEPPKSVAFIQCVGSRDKTV 392

WP_048037482 313 EEIEFEAGAVIVSTGYQLFDASRKKEYGFGKYPDVITNMQLERMLNSAGPTGGRVLVPSTGQPPESVAFIQCVGSRDKTV 392

WP_011307542 313 EEIEFEAGAVIVSTGYQLFDASRKKEYGFGKYPDVITNMQLERMLNSAGPTGGRVIVPSTGKPPKSVAFIQCVGSRDKTV 392

WP_023843920 313 EKIEFTVGAIILSTGYSHFDASRKPEYGYGIYPDVITNMELERLLNAAGPTKGRVLSPSTLKVPEKVSFIQCVGSRDEQV 392

WP_015324480 313 ETISIKVGAIILASGYKIFDAARKEEYGYGVYPDVITNMELERLLNASGPTRGKVLVPSTRQIPKKVAFIQCVGSRDETV 392

WP_048194718 313 EEFSFTVGAVIVATGYQGFDAKRKEEYGYSVYPDVLTNMELERLLNASGPTRGRVVVPSTHETPEKVAFIQCVGSRDETV 392

WP_013897960 313 EEFIFNAGAIIVSTGYSLFDASRKEEYGYGIYPDVITNMELERLLNASGPTRGRVVVPSSGKVPERVAFIQCVGSRDESV 392

WP_013194860 314 EEFSFDAGAVIVATGYNVFDASRKEEYGYSKYPDVITNMELERMINASGPTHGKVVVPSTKEIPQKVAFIHCVGSRDETV 393

WP_013037418 313 TQFQLNVGAIIIATGYRLFDASRKEEYGYGIYPDVMTNMELERLLNASGPTRGRVTCPSTGEVAEEVAFIQCVGSRDESV 392

WP_048089472 312 EGFEFNVGAIILATGYQPFDARRKEEYGYGRYRNVVTTLELERMLSAAGPTHGRVISPSTGADVKSAAFILCVGSRDEQV 391

WP_014586170 313 EEFRFDVGAIIVATGWQSFDPARKEEYGYGRYRDVISALQVERLLNAAGPTGGEVVRPSTGEVAKSVAFLQCVGSRDETV 392

WP_042684393 310 STEEVEVGAIIVATGYSTFDPARKPHYHFSEGKDVITSAQLERMLSASGPTGGTLVQPSSMSVPKSVAFVQCVGSRDEQV 389

WP_012964674 313 EDLEIEAGVIIVATGYKPFDARRKEEYGYGVYKNVITTLELERLLSASGPTLGNLYRPSDSSVPRKIAFIQCVGSRDVKT 392

WP_010878733 313 ETLEIKAGAIIVATGYKIFDARRKTEYGYGRFKNVITTIELERLLSASGPTMGRLLRPSDSTVPKRIAFIQCVGSRDEKT 392

KPL16356 325 ELIDLDVGTIIVATGVDVYDPTEMTEYGYRRYENVITSLEFERLINAGGPSGGHLIRPSDKKIPKTVGFIQCVGSRNEKR 404

KPV63986 334 ETVELEVGTIIVAAGADVYDPSSLANYGYGKFPNVITSLEFERLINAGGPSGGHLIRPSDMQIPKSVAFIQCVGSRSEK- 412

WP_051309217 325 EELSFEVGTIIMATGMDIYDPTRLDEYGYTRFDNVLTSMEFERLINAGGPTGGEVVRMTDRKRPRSVAFIQCVGSRSLHK 404

WP_041286569 321 QILELEVGTIVVATGVEVYDPTALTELGYGKFPNVITTLEFERLINAGGPSGGELIRPSDRKRPKKVAFLQCIGSRSKR- 399

KPJ88130 330 SEETFRVGTIVVCTGVDVFDASEIPQYQYRRAPNVITSLEFERMINAGGPTHGHLIRPSDHRIPKSVAFIQCVGSRSNK- 408

*** ****

WP_011022820 393 GNEYCSRVCCMAALKNSQMVKERYPDTDVTIHYIDIRAAGEMYEEYYTRTQEMGVDFIRGKVAEV YSGEDGRPVVRF 469

WP_048037482 393 GNEHCSRVCCMAALKNSQMVKERYPGTDITIHYIDIRAAGEMYEEYYARTQGMGVDFIRGKVAEV YAGEDGRPVVRY 469

WP_011307542 393 GNEYCSRVCCMAALKNSQMVKERYPDTEITIHYIDIRAAGEMYEEYYTRTQSMGVDFIRGKVAEI YAGEDGRPVLRY 469

WP_023843920 393 GNPYCSRVCCMSSMKNAQLLKERYPDVDITIHYIDVRASGEMYEEYYIRSQEMGINFVRGKVGEI LQDFDGKLKLRY 469

WP_015324480 393 GNPYCSRVCCMSAMKNAQLLKERYHDIEIVIHYIDIRAAGEMYEEYYIRSQSMGIDFIRGKVAEV QQDLNNKLFMRF 469

WP_048194718 393 GNPYCSRVCCMSSMKNAQMIKERYPDTDVTIHYIDIRAAGEMYEEYYVRSQMMEINFIRGKVAEV QLDPQGQMQLRY 469

WP_013897960 393 DNPYCSRVCCMASMKNAQLLKERYPQIEIVIHYIDVRASGEMYEEYYTKTQSMGIDFIRGKASQV LMDKQGRPALRF 469

WP_013194860 394 GNPYCSKVCCMSAIKNAQLLKERYPEMDIRLHYIDIRASGEMYEEYYTNAQSMGIQFVRGKVAEI QEDNSGKPVLRY 470

WP_013037418 393 GNPYCSRVCCMSAMKNAQLLKERYPDIRITIHYIDIRASGEMYEEYYTKTQAMGVDFVRGRVAEI VQEHDASLSLRY 469

WP_048089472 392 GNPYCSRVCCMASVKNAMKIAEKYPDAKVSVHYIDIRAAGEMYEEYYRRAQEMGVSFVRGRVAEV -EESEGKTIIHY 467

WP_014586170 393 GNAYCSRVCCMYALKNAQLIKEKYPETEVSIHYIDLRAGGEGYEEFYMRAQRLGINFIRGRVSEV -EEVDGSLRVNY 468

WP_042684393 390 GNAYCSRVCCMSAIKNALQIKERHPEVEVSVHYLDVRACGEGYEEMYLRAQKAGVRFIRGLPGEI[4]LEGRDGGADVLY 470

WP_012964674 393 -NKYCSRVCCMVSIKNAYIIKERYPEADVSVFFIDIRAFGRMYEEFFARAQEKGIRFIRGRVAEI YELENGNLILTY 468

WP_010878733 393 -NKYCSRVCCMVSLKNAYAIKERYHDAEITIFYIDIRAFGRMYEEFYRRVQEAGVRFIRGKVGEI IENENGNLIVSY 468

KPL16356 405 GNPYCSNVCCMNTIKDSLLIMEHWPDTKIKIFYLDIRAFGKGFEDLYKRSKSSGVMYIRGLPAEI[1]EDPVTNNLVIVS 482

KPV63986 413 GHLYCSNVCCMNTIKDSLLIKEHWPDTQIHVFYVDIRAYGKGFEDLYKRAKKEGVTFIRGLPAEI[1]EDRKSHNLWLIG 490

WP_051309217 405 GSSYCSNVCCMNTIKCAFMLKEHYPDIEVKVFYIDIRAFGKGFEDQYRRTKRMGVQYIRGLPGTV[1]ENTDNGNLTVFV 482

WP_041286569 400 SNPYCSNVCCMNTVKDALLIKEHWPDTEIHVFYIDIRAFGKGFEDLFQRARREGVVFMRGIPGEI[1]EDIQTGDLTLLG 477

KPJ88130 409 TNPYCSNVCCMNTIKDSLLIKEHWPDTEIKVFYIDIRAFGKGFEDLFQRAKRLGVQFIRGLPGEI[1]Q-LNSGNLRLIG 485

**#**

WP_011022820 470 ENTLESSVEEEAHDLVVLSTGYEPTKAAEGIGRMLNLARRPDRFFASAHPKMRPVDAPVSGVFLAGCASGPKEIQVSIAQ 549

WP_048037482 470 ENTLESRVEEEAHDLVVLSTGYEPSKAAEGIGRMLNLARRPDRFFASAHPKMRPVDAPVSGVFLAGCASGPKEIQVSIAQ 549

WP_011307542 470 EDTLESRIEEEACDLVVLSTGYEPSKSAEGIGRMLNLARRPDRFFASAHPKMRPVDAPVSGVFLAGCASGPKEIQVSIAQ 549

WP_023843920 470 EDTLSSELFEETTDLVVLATGMENVKDADKISRVLNLTRRTDRFFSIAHPKMRPVDSHVKGIYIAGCASGPKEIQVSIAQ 549

WP_015324480 470 EDTLSSEVTEEYYDLVVLSTGMEAPDDADKISRVLNLSRRTDRFFAIAHPKMRPVDSHVKGIYIAGCASGPKEIQAAIAQ 549

WP_048194718 470 EDTLECAIREEPYDLVILATGMEASTTTEPIAKMLNLSKRTDRFLSIAHPKMRPVDAHINGVFIAGCASGPKEIQAAIAQ 549

WP_013897960 470 EDTLESEIRQESYDMIVLATGMEAPENSRTITRMLNLSCRADRFYSIAHPKMRPVDSHIDGVFIAGCATGPKDIQSSIAQ 549

WP_013194860 471 EDTLENKIFEETYDLVVLSVGLEANKDEK-ISSMLNLSTRSDGFYTVSHPKMRPVESNIDGVYIAGCASGPKEIQISIAQ 549

WP_013037418 470 ENTLESRIEEKQYDLVILSTGMEADPSAKPIGSMLKMAKRPDRFFAVSHPKMKPVDSHINGVFLAGCASGPKEIQVSIAQ 549

WP_048089472 468 EDTLSGDTRHEAYDLVVLAIGMEANMDAGNIGRMLNLSTRPDRFFQSAHPKMRPVQTHTKGVFIAGCAGGPKEIQVSIEQ 547

WP_014586170 469 EDTLLGGFRSKPYDLVVLSAGLEPNKDADVVGNLLGLAKRPDGFFEIAHPKMRPVEAHIEGVFIAGCASGPKEIQVSIAQ 548

WP_042684393 471 EDTLAGRLMRTHYDLVVLSVGMESPEEAEPIARMLNLRRRDDRFLAVAHPKMRPAESVYRGIFLAGCATGPKEIQLSITQ 550

WP_012964674 469 ENTLTGEIKEEEFELVVLSIGMEGNTD---LANKLGISVGEDGFYDVAHPKLRPAETDVKGIFLAGAASGPKDIQDSVAS 545

WP_010878733 469 ESTLEGEVREEEFDLVVLSIGIEGNRD---VATKLGLGIGEDGFFEVAHPKLRPAETNVKGIFLAGCASGPRDIQDSVAS 545

KPL16356 483 ENTNTGKVEEHEVDMAILSVGLIPTKDSDVIQRLFTLSKTSDGFFMESHPKLKPVDAPTQGVFFAGCAEGPKDIKDSVTQ 562

KPV63986 491 ENTLQKELYRVNVGMVILSIGIESRRDSEVIQRLLTLSRNPDGFFMEAHPKLRPVDTPTGGVFLAGCAEAPKDIKDSVTQ 570

WP_051309217 483 ENPETRIVERHELEMLVLAVGVRPPSGLKHLQEMLALQRNPDGFFLEAHPKLQPVDAATRGIFFAGCSEGPKDVKDSVTQ 562

WP_041286569 478 ENTLLGSHYKFHMDMVILSVGIKPHKDAEKIQRLLNLATDTDGFYMEAHPKLRPVDTTTGGVFLAGAAEGPKDIKDSVTQ 557

KPJ88130 486 EMTLMDRLYNMETEMVILSIGLKPSIGSDVVRKFLTMSLTSDGFFMVAHPKLRPVDTTSRGVFLAGCAEGPKDIKESVTQ 565

*** * * * * * * ***

WP_011022820 550 GSACASKVMQLLGTGELEADPMGAHVDPDKCIGCRTCVEVCKFGKISI--ENK-KAVVDEVSCYGCGDCSAACPVGAIQM 626

WP_048037482 550 GSACASKVMQLLGTGELEADPMGAHVDPDKCIGCRTCVEVCKFGKISI--VDK-KAVVDEVSCYGCGDCSAACPVGAIQM 626

WP_011307542 550 GSACASKVMQLLGTGELEADPMGAHVDPEKCIGCRTCLEVCKFGKIKI--ENK-KAVVDEVSCYGCGDCSAACPAGAIQM 626

WP_023843920 550 GSGAAAKAMQLLSKGELEMDPLSAHVNPDKCIGCGICADTCKFNKITM--VDR-KAVVDELSCMGCGACSAACPADAIWM 626

WP_015324480 550 GLAASAKTMQLLTWGELETDPLSAHVDEEKCIGCKICEDVCQFGKIKV--ING-KATVDEVSCYGCGACSASCPTDAIMM 626

WP_048194718 550 GSATAARSTRLLAKGELKNDPFSAHVDPEKCIGCRICESVCNFNTINV--IDG-KAVVDEISCQTCGSCSASCPTDAITM 626

WP_013897960 550 GCAAAAKVMKLLGSGELEADPLCAVVEKASCVGCGLCEDVCIFGKIRV--IDS-KAVVDEISCQGCGTCSAACPTDAIDM 626

WP_013194860 550 GSAVASKAMQLLSRGELETDPMSAFVDTDKCTGCGICQDVCKFGKIKL--YNH-KAEVDELSCHGCGSCSAACPEDAIYM 626

WP_013037418 550 GSAAASRAIRLLHPGELRTDPLSAHVDYDTCIGCGVCTDVCDYGTIKI--ENG-KAFVDEVSCHGCGTCSAACPVDAISM 626

WP_048089472 548 GSAAAAKAESLLHKGEIELDPMSAYVITELCDGCRICEAVCEFGRIRVt--DG-KASVDEVACRGCGPCAAACPNGAVQL 624

WP_014586170 549 GEAAAAKAMRLLVRGELALDPVVAVVDQEKCIGCKLCVDTCPGKAISV---NG-TALVDEAACKGCGTCAAACPVDAIDM 624

WP_042684393 551 AGEAASRAMSLLSKGVIEADAYTAVVDAEKCIGCGICESVCPSASIQLggNPK-RARVDGSTCIACGTCIASCPADAIDQ 629

WP_012964674 546 AGLAASKAMELIFGGEAEFDPYNAYVNEEKCIGCRICEEVCNFNAVTF--ENK-KAKIDPNACVMCGVCAASCPADAIDL 622

WP_010878733 546 AGLAAAKAAQLVLTGETEFDPYNAYVDEEKCIGCRICEKVCEFNAVTV--D-R-KAKINPNACAMCGICVAACPADAIDM 621

KPL16356 563 ASAAASRAGILMKAGKIKVEAITSVIDEDICNACGLCARVCPYNAIEVdpKNKiPAHVIEAACAGCGTCGAECKHGAITM 642

KPV63986 571 ASAAAARASTLMAKGSVTVEAITPRVIIENCKACVLCAKVCPYNAIFVnkELK-RAEVIEAACAGCGTCGAECPFDALIM 649

WP_051309217 563 ASAAAMRASILMSKGMLSVEGITAEVNPELCSSCGLCAKVCPYKAITVdrIAKsGAHVTTAACSGCGTCSAECPENAIEM 642

WP_041286569 558 ASAAASRANILMSKGEVQIPAITSHIDPEKCTACGLCARVCPYHAIEGskEQG-FYRVIEAACQGCGACVPECRFGAIDQ 636

KPJ88130 566 ASAAAARANIIMNRGKITVEAITARVQEDLCTGCGACTKVCPYNAIYLn-QNK-RAVVIDAACAGCGTCSAECQYGAIIM 643

*** * ***

WP_011022820 627 RNFENEQILAQVREA**TAHKSQCPFIVAFLCNWCSYACADLTGMSRIRYPTNIRVIRTMCSARVNPEFVLEALKGGADGVL** 706

WP_048037482 627 RNFENEQILAQVRAA**TAHKSQCPFVVAFLCNWCSYACADLTGMSRIHYPTNIRVIRTMCSARINPEFVLEALKGGADGVL** 706

WP_011307542 627 RNFENEQILAQVREA**TAHKSQSPFIVAFLCNWCSYACADLTGMSRLHYPTNIRVIRTMCSARVNPEFVLEALKGGADGVL** 706

WP_023843920 627 RNSTDAQIVAQIHAA**TEVKSESPLIVAFLCNWCSYTCADLAGTSRIQYPTNIRVIRVMCAGRVDPSFVLEALERGADGVL** 706

WP_015324480 627 RNSTDEQILAQVRAA**TEVKSEFPLIVAFLCNWCSYTCADLAGVSRIQYPTNIRAIRVMCAGRVDPDFVLEAFKGGADGVL** 706

WP_048194718 627 PHSTDEQIIAQIRAA**VEIKDEFPLIIAFLCNWCSYGSADLAGTSRIQYPTNVRIIKVMCAGRVDPDFVLEALQGGADGVL** 706

WP_013897960 627 RHFTDEQIFAQIEAA**VESRDEFPLIIGFLCNWCSYSCADLAGVSRIDYSTNIRIIRTMCAGRVDPEFVIKALEGGADGVL** 706

WP_013194860 627 RNQTDAQIHSQIEAA**LEVKDEFPLIVSFLCNWCSYACADLAGTSRIQYPTNVRIIRVMCAGRVDPEFVLTAFEKGADGVM** 706

WP_013037418 627 HNHTDEQVRAQIKAA**LEVKDEFPLIVAFLCNWCSYASADLAGTSRIQYPTNVRIIKVMCAGRVDPDFVMEAFEMGADGIF** 706

WP_048089472 625 RSYTDEQIMAQIEEA**TRDINEYPLVIGFLCHWCSYAAADLAGSMRIQYPTNLRNIRVLCTGRINPSFVLEALKRGADGVL** 704

WP_014586170 625 TLFSDEQIMAQVRAA**TAVKGQYPFIVGFLCNWCSYAGADLAGTSRIQYPTNMRAIRVMCAGRVDPAFVLEALKGGADGVL** 704

WP_042684393 630 KNFTDSQILSQIRAI**-DPESEYPLIVAFLCNWCAYGAADLAGISKLKYPPNVRIIRVMCSGRVDPQMVLEALKCGADGVL** 708

WP_012964674 623 GFFKEDAIVAMIDAL**AEEKKVEPLILIFACHFCSYGALDLAGTTKTQYSPNVRVIRTLCSGRVDPEWILRALKRGIDGVM** 702

WP_010878733 622 GFFSDEGIKAMIDAL**GEEKNADPLALAFACWYCSYGAADLAGTTKVQYEPNVRIIRVLCSGRVDPEWVLRALARGIDGVI** 701

KPL16356 643 RHFTDQQILAQVDAV**TEE-NADKKIVAFCCNWCSYAGADFAGVSRMQYPPAVRIIRSMCSGRISEEFVLHAFRRGAAAVL** 721

KPV63986 650 RHFTDEQIYAQIDAA**TEQ-DADTKIVAFCCNWCSYAGADFAGVSRMQYPTNVRIIRTMCSGRVAPKFVERAFARGAAAVL** 728

WP_051309217 643 HHFTDPQIEAQIEAV**LGD-RAEERVLVFACNWCSYAGADFAGVSRLEYPASTRLIRTMCSGRVDEKFLWKAFALGVPAIL** 721

WP_041286569 637 AHFTEEQIVSQIDAA**LAI-DPHNKILAFACNWCSYAGADFAGVSRMQYPHNVRIIRTMCSARVSPSWIEKAFSMGAGGVL** 715

KPJ88130 644 RHFTDDQIMAQIDAF**TEH-EPEKKILAFNCNWCSYAGADFAGIGRMQYSPEVRIIRTMCSGRVSSRFVEYAFARGVAMVL** 722

*** ***

WP_011022820 707 **VAGCRMDECHYIHGNFDAKKRMDILKEVIKEIGLDPKRLRTLWISAAEGERFSNTINEFVKELEE-IGPIGSEFKQECA[ 9]** 793

WP_048037482 707 **VAGCRMDECHYIHGNFDAKKRMDVLKEVIKEIGLDPKRLRTLWISAAEGERFSNTITEFVKELEE-IGPIGSELKQEYT[ 9]** 793

WP_011307542 707 **VAGCRMDECHYIHGNFDAKQRMDVLQEVLKEIGLDPRRLRTLWISAAEGERFSNTITEFVKELEG-IGPIGTEFKLEET[ 8]** 792

WP_023843920 707 **VAGCRLGECHYIFANYNAKQRMEALKEVLGDVGIDPGRLSVEWISASEGERFANSIEGFVDYLKK-IGPIGSELKEAEQ** 784

WP_015324480 707 **VAGCRLGECHYIFANYSAKKRMEVLQGVLEDIGIDPHRLKVEWISAAEGERFARSIESFVDELKE-IGPIGSELLEGHN[ 2]** 786

WP_048194718 707 **VTGCRLDECHYILGNIDAKHRMENLKEVLDEIGLDPRRLRLQWISAAEGDKFAKTIEDFVDELTE-LGPVGSELPEEQE** 784

WP_013897960 707 **VAGCKLGECHYVHANYSAKKRLEALQNVLEETGIDPSRLRLLWISASECEKFAKTVDDFVDNLKN-IGPVGHEILEAAD** 784

WP_013194860 707 **VSGCRLGECHYVYGNYNAKQRMDVLKDVLEEIDINPNRLNVEWISASECNRFAESIENFVEELEK-IGPIGSEILEAPN[ 5]** 789

WP_013037418 707 **VAGCRLGECHYVHGNYHAQTRMENLGETLSKEGFDSGRLRVEWISAAEGEKFASTIEDFVDYLKA-IGPIGSELEEADR** 784

WP_048089472 705 **VAGCRLGECHYTIGNYCARQRMDVLGKLLADMGLNESRLRVEWIAASEGERFACIVKDFVKQLKE-IGPIGSELKR---** 779

WP_014586170 705 **ISGCRLGECHYNKGNYQAYQRVQVLRGVLEKVGINPGRVKIIWCAASEGEILAKEVRKFVSELKE-MGPAGSELAALRL[ 8]** 790

WP_042684393 709 **IGGCRMGECHYQTGNCHCEARMEALREMLAEQGISPDRIATVWVSANESQTLIEALETLIERVEQ-LGPVGKEMDGGWE[ 7]** 793

WP_012964674 703 **VTGCRLGECHFRVGNYHAVDRIKALKKLLEEIGINPERVETSWHSAGEGAEIAKDIDEFVERIAK-LGSIYEEVAR---** 777

WP_010878733 702 **IAGCRLGECHFKYGNYKAKDRFEALKEALKEVGIEPERVRCIWHSAGEAEGIANDFNEFVEELKK-LKS----------** 769

KPL16356 722 **VAGCHLNDCHYIDANYQTLKRVEKLWKKLEKMGIEKERLQLAWISAAEGEKFASKIKQMKEIIDG-VSA--GEIKK---[ 6]** 800

KPV63986 729 **VSGCHLGDCHYINANYQTQKRVERLWKKMEKLGLNKDRLQLLWVTAAEGERFASKIKEMQGIVNS-VSQ--EETEKGKN[15]** 819

WP_051309217 722 **VSGCHLSDCHYIDANHWTEKRIHRMWRKMEKLGVRQERLQLEWISAAEGIRFQEAMQKM-EGIRKtVSS--EEIALTR-[16]** 812

WP_041286569 716 **VSGCHPADCHYNNANQNTARRVDRFWKRMEKLGINKDRLRLAWVSAAEGAQFAKVIKEMEEGLRK-LTP--QEIEEAA-[13]** 803

KPJ88130 723 **ISGCHIGDCHYIDTNTYTEKRYNQVQKIMEHGGLDQERLQLVWVSASEGQIFQEKVNEMKRKLKN-LSM--DEIKRGML[26]** 824
