# Supplementary figures and images for: A Ferredoxin- and F420H2-Dependent, Electron-Bifurcating, Heterodisulfide Reductase with Homologs in the Domains Bacteria and Archaea
Source: mBio. 2017 Feb 7;8(1):e02285-16. doi: 10.1128/mBio.02285-16 (PMC5296606; doi:10.1128/mBio.02285-16)

**Figure S2.**


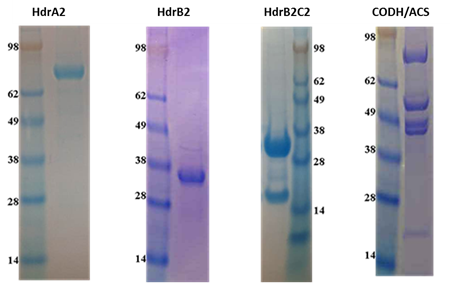

Supplement: FIG S2 [file mbo001173173sf2.docx]

**Figure S4.**


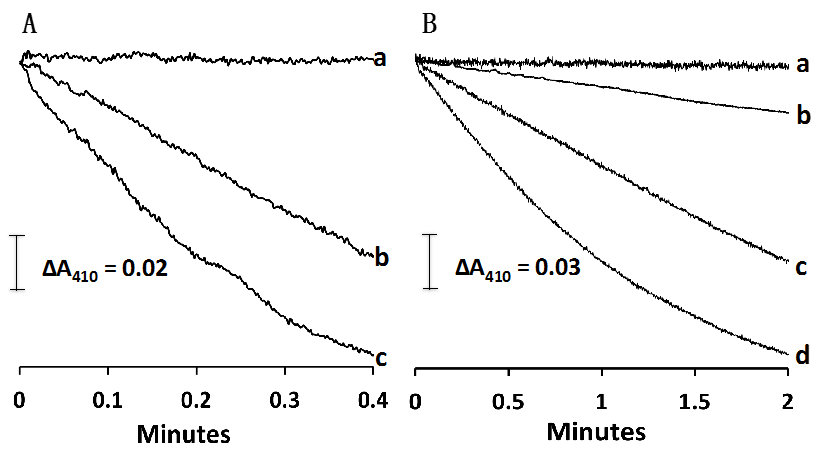

Supplement: FIG S4 [file mbo001173173sf4.docx]

**Figure S5.**


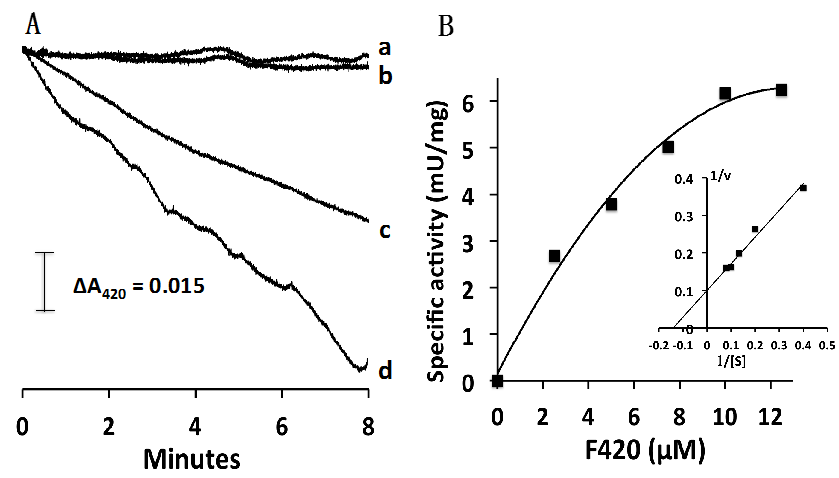

Supplement: FIG S5 [file mbo001173173sf5.docx]
