# Supplementary material for: A Ferredoxin- and F420H2-Dependent, Electron-Bifurcating, Heterodisulfide Reductase with Homologs in the Domains Bacteria and Archaea
Source: mBio. 2017 Feb 7;8(1):e02285-16. doi: 10.1128/mBio.02285-16 (PMC5296606; doi:10.1128/mBio.02285-16)
Supplement: FIG S3 [file mbo001173173sf3.docx]

**Figure S3.**

**A**

M.t. HdrA 1 maeekketmeePKIGVYVCHCGVNIGGVVDVEAVRDYAAKLPNVVIAKDYKYYCSDPGQLEIQKDIKELGINRVVVAACS 80

M.a. HdrA2 1 -----------MRIGVYICHCGLNIAGVIDVSALEAMANELEDVVLAREVQFLCSDSGQEGIIKDIKDNKLDRVVVAACS 69

M.t. MvhD --------------------------------------------------------------------------------

M.t. HdrA 81 PRLHEPTFRRCVEEAGLNQFLFEFANIREHDSWVHMDNPEGATEKAKDLVRMAVAKARLLEPLEASKVSVDDKALVIGGG 160

M.a. HdrA2 70 PRLHEKTFRHVMEKAGLNPYLMEMVNIREQCSWVHADDPQMATQKAFDLIRMGVAKARFLRELSATNSKASRNVLIIGGG 149

M.t. MvhD --------------------------------------------------------------------------------

M.t. HdrA 161 VAGIQAALDLADMGFKTYMVEKRPSISGRMGQLDKTFPTLDCSMCILAPKMVDVGKHDNIELITYAEVKEVDGYIGNFKV 240

M.a. HdrA2 150 VAGIEAALNLAEAGFPVTMVEKESTIGGKMALMNEVFPTNDCSICVLAPKMTEVQNHPNITLYTYSEVTDISGSVGKFHV 229

M.t. MvhD --------------------------------------------------------------------------------

M.t. HdrA 241 KIEKKPRYIDEELCTGCGS-CVEVCPIEMPNYFDEGIGMTKAVYIPFPQAVPLCATIDKDYCIECMLCDEVCERGAVKHD 319

M.a. HdrA2 230 RVKRKPRFVLEDKCKGCVDlCSGVCPVEIENPMNYGIGKTRAIYMPIPQSVPQVVLIDPDHCVGCGLCQLACPAEAVDYE 309

M.t. MvhD --------------------------------------------------------------------------------

M.t. HdrA 320 QEPEEIEIEVGTIIVATGYDAYDPTEKLEYGYGRHTNVITGLELERMINASGPTDGKVLKPSDGEKPKRVAFIHCVGSRD 399

M.a. HdrA2 310 QKPEEIEFEAGAIIVSTGYQLFDASRKKEYGFGKYPDVITNMQLERMLNSAGPTGGRVLVPSTGEPPKSVAFIQCVGSRD 389

M.t. MvhD --------------------------------------------------------------------------------

M.t. HdrA 400 EQIGKPYCSRVCCMYIMKNAQLIKDKMPDTEVTLYYMDIRAFGKGFEEFYKRSQEKyGIKFIRGRPAEVIENPDLTLTVR 479

M.a. HdrA2 390 KTVGNEYCSRVCCMAALKNSQMVKERYPDTDVTIHYIDIRAAGEMYEEYYTRTQEM-GVDFIRGKVAEVYSGEDGRPVVR 468

M.t. MvhD --------------------------------------------------------------------------------

M.t. HdrA 480 SEDTLLGKVTEYDYDMVVLGVGLVPPEGAETLRQTIGLSKSADGFLMEAHPKLRPVDTLTDGVYLAGVAQGPKDIPDAVA 559

M.a. HdrA2 469 FENTLESSVEEEAHDLVVLSTGYEPTKAAEGIGRMLNLARRPDRFFASAHPKMRPVDAPVSGVFLAGCASGPKEIQVSIA 548

M.t. MvhD --------------------------------------------------------------------------------

M.t. HdrA 560 QASGAAARAAIPMVKGEVEIEPIIAVTDSDVCGGCEVCIELCPFGAISIEEGHANVNVALCKGCGTCVAACPSGAMDQQH 639

M.a. HdrA2 549 QGSACASKVMQLLGTGELEADPMGAHVDPDKCIGCRTCVEVCKFGKISIENKKAVVDEVSCYGCGDCSAACPVGAIQMRN 628

M.t. MvhD --------------------------------------------------------------------------------

M.t. HdrA 640 FKTEQIMAQIEAALNEPASK------------------------------------------------------------ 659

M.a. HdrA2 629 FENEQILAQVREATAHKSQCpfIVAFLCNWCSYACADLTGMSRIRYPTNIRVIRTMCSARVNPEFVLEALKGGADGVLVA 708

M.t. MvhD 1 -------------MAEDDIK--IVMFCCNWCSYGGADTAGTARMQYPTNIRVIRVMCSGRIEPQFVLKAFREGADGVLVT 65

M.t. HdrA --------------------------------------------------------------------------------

M.a. HdrA2 709 GCRMDECHYIHGNFDAKKRMDILKEVIKEIGLDPKRLRTLWISAAEGERFSNTINEFVKELEEIGPigSEFKQECAVPgv 788

M.t. MvhD 66 GCHHGDCHYDAGNYKLDRRMRLIYKLADELGIGRERIHHDWISASEGEKFAETVKMMVNRIKGLGP--SPIKKQLAEA-- 141

M.t. HdrA -----

M.a. HdrA2 789 eevtq 793

M.t. MvhD -----

**B**

M.t. HdrB 1 M-EIAYFLGCIMNNRYPGIEKATRVLFDKLGIELKDMEGASCCPAPGVFGSFDKTTWAAIAARNITIAEDMGADIMTECN 79

M.a. HdrB2 1 MAKLSLFRGCIVPNRYPGIEKATKLCLQKLEVDAVDLPGASCCPAPGVFKSFDKATWLALASRNIVLSERMGRDILTVCN 80

M.t. HdrB 80 GCFGSLFETNHLLKEDEEMKAKINEILKETGREYKGEVNVRHFAEVLYNDVGLDKLSELVEKPLNLNVAVHYGCHFLKPS 159

M.a. HdrB2 81 GCYGSLADANIELKNDPEMKACTNSCLKEIGMEYKGTAEVRHIIEFLYKELGPEKLKSFITTPLDLKVALHYGCHLIKPS 160

M.t. HdrB 160 DEINIDNPERPTILDEIVEVTGAKSVEYKDKMMCCGAGGGVRSRDLDVALDFTREKLTNMKEAGVDAIVNVCPFCHLQFD 239

M.a. HdrB2 161 KERNLGETEAPVFFDELVEATGAKSVDYTDKMMCCGAGGGVRSGHAAESLEMLEHKLACIRNAGVDCIVNACPFCHLQFD 240

M.t. HdrB 240 VGQMEIKDKFGEEFDIPVLHLAQLLGLAMGLPKEDLVVDAHQVCVDECLEKLEELDRLAPGSG 302

M.a. HdrB2 241 RGQLAVNEKFGTDYSIPVLHYSQLLGLALGFSPDELGIEQNAIQNIEFLAKIYEI---SAGLR 300

**C**

M.a. HdrC2 --------------------------------------------------------------------------------

M.t. HdrC 1 MKTMSVLKRLKEMLMGEKKEEDSKTEKPVETETPSEPIKETEKPQPEEKPSAEPSSSASETSDAPEEPVKVSEAPEAEET 80

M.a. HdrC2 1 ---------------------------------------------------------------------------MSEEL 5

M.t. HdrC 81 VKVPEAPEKETADADEVETEEPAETSDEDEEVVEDETSSEDSDSDEESEDEEEKSERSDIMTLLQREENIIRKGNIDKEF 160

M.a. HdrC2 6 LKLLKAEGLDLLS-CMHCGICTGSCPSGRHTGLNTRRIIRDARKN-RAAVLSDYDLWLCTTCYTCQERCPRGIPITDAIL 83

M.t. HdrC 161 SEKIKAAGGDSLEYCFQCGTCTGSCPSGRRTPYRVRQIIRKANVGLKDEIISDPALWMCTTCYSCQERCPRKVKIVDVVK 240

M.a. HdrC2 84 ELRRLAVREGLMLPEHRFVSEMVLECGHAVPLDEETKKKREELGLDPIPETVQKDPEALEGLKTLLKTCKFDELVAKK-- 161

M.t. HdrC 241 LARNEAAKAGFMAPAHKAVGSFVIKTGHGVPINDATMELRKAVGLGELPPTTHQFPEALEEVQKIIKATGFDQLIGYNWE 320

M.a. HdrC2 -----

M.t. HdrC 321 TGELE 325
