# Supplementary material for: A Ferredoxin- and F420H2-Dependent, Electron-Bifurcating, Heterodisulfide Reductase with Homologs in the Domains Bacteria and Archaea
Source: mBio. 2017 Feb 7;8(1):e02285-16. doi: 10.1128/mBio.02285-16 (PMC5296606; doi:10.1128/mBio.02285-16)
Supplement: TABLE S1 [file mbo001173173st1.docx]

Table S1. Primers used for amplification of genes and the construction of plasmids.

| plasmid | protein | primer sequences |
| --- | --- | --- |
| pET22b-*ma0431* | Fdx | Forward 5’-GAGATATACATATGCCAGCAATAGTTAATGCAGAT-3’ |
|  |  | Reverse 5’-GTGGTGGTGGTGGTGCTCTTCTACCTTAAT-3’ |
| pET22b-*ma2868* | HdrA2 | Forward 5’-GAGATATACATATGCGAATCGGAGTCTACATTTGC-3’ |
|  |  | Reverse 5’-GTGGTGGTGCTCGAGCTGTGTCACCTCCTCAACC-3’ |
| pET22b-*ma4237* | HdrB2 | Forward 5’-GAGATATACATATGAGTGAAGAACTGCTAAAACTG-3’ |
|  |  | Reverse 5’-GTGGTGGTGGTGGTGTTTTTTAGCCACCAGCTCGTCG-3’ |
| pETDuet-*ma4236*-*ma4237* | HdrC2 | Forward 5’-CACCACCACCACCACCACTG-3’ |
|  |  | Reverse 5’-CATATGTATATCTCCTTCTTAAAGTTAAACAAAATTATTTC-3’ |
|  | HdrB2 | Forward 5’-CATGGTATATCTCCTTCTTAAAGTTAAACAAAATT-3’ |
|  |  | Reverse 5’-TAATGCTTAAGTCGAACAGAAAGTAATCGTATTG-3’ |
