# Supplementary material for: A Ferredoxin- and F420H2-Dependent, Electron-Bifurcating, Heterodisulfide Reductase with Homologs in the Domains Bacteria and Archaea
Source: mBio. 2017 Feb 7;8(1):e02285-16. doi: 10.1128/mBio.02285-16 (PMC5296606; doi:10.1128/mBio.02285-16)
Supplement: TABLE S2 [file mbo001173173st2.docx]

Table S2. Standard Gibbs free energy values for reactions in the Fe(III)-dependent ANME pathway proposed for *Methanosarcina acetivorans*.

| **Enzyme**  **(reaction**  **Number)*** | **Reaction** | **ΔG°′** |
| --- | --- | --- |
| Mcr (1) | 2 x {(1) CH_4_ + (1) CoMS-SCoB → (1) CH_3_-SCoM + (1) HSCoB} | 2 x (+31.26) † |
| Mtr (2) | 2 x {(1) CH_3_-SCoM + (1) THSPT → (1) CH_3_-THSPT + (1) HS-CoM} | 2 x (+29.23)† |
| HdrDE  (3,4) | 2 x {(1) HS-CoM + (1) HS-CoB + (1) MP  → (1) CoMS-SCoB + (1) MPH_2_} | 2 x (-31.37)† |
| Cyt *c* (5,6) | 2 x {(1) MPH_2_ + (2) Fe^3+^ →(1) MPH_2_ + (2) Fe^2+^} | 2 x (-117.13)§ |
| CODH/ACS (7) | (1) CH_3_-THSPT + (1) CO_2_ + (1) HS-CoA + (2) Ferredoxin*_red_* + (2) H^+^  → (1) CH_3_-SCoA + THSPT + H_2_O + (2) Ferredoxin*_ox_* | -40.45† |
| Pta, Ack (8) | (1) CH_3_-CO-SCoA + (1) HPO_4_¯ + (1) ADP  → (1) ATP + (1) HS-CoA + (1) CH_3_COO¯ | -5.26† |
| Mer, Mtd, Mch  (9) | (1) CH_3_-H_4_HPT + (2) F_420_  → (1) CHO-H_4_HPT + (2) F_420_H_2_ | -14.72† |
| Ftr, Fmd  (10) | (1) CHO-H_4_HPT + (2) Ferredoxin*_ox_* + (1) H_2_O  → (2) Ferredoxin*_red_* + (1) H_4_HPT + (1) CO_2_ + (2) H^+^ | -32.03† |
| HdrA2B2C2 (11) | (2) F_420_H_2_ + (2) Ferredoxin*_ox_* + (1) CoMS-SCoB  → (2) F_420_ + (2) Ferredoxin*_red_* + (1) HS-CoM + (1) HS-CoB + (4) H^+^ | -38.59¶ |
| HdrDE  (12,13) | (1) HS-CoM + (1) HS-CoB + (1) MP  → (1) CoMS-SCoB + (1) MPH_2_ | -31.37† |
| Cyt *c* (14,15) | (1) MPH_2_ + (2) Fe^3+^ → (1) MPH_2_ + (2) Fe^2+^ | -117.13§ |
| Rnf (16,17) | (2) Ferredoxin*_red_* + (2) Fe^3+^ → (2) Fe^2+^ + (2) Ferredoxin*_ox_* | -249.30‡ |
| **Overall** | $\left( \boldsymbol{2} \right)\boldsymbol{C}\boldsymbol{H}_{\boldsymbol{4}}\boldsymbol{+}{\left( \boldsymbol{1} \right)\boldsymbol{H}}_{\boldsymbol{2}}\boldsymbol{O+}{\left( \boldsymbol{8} \right)\boldsymbol{Fe}}^{\boldsymbol{3+}}\boldsymbol{+}\left( \boldsymbol{1} \right)\boldsymbol{HP}\boldsymbol{O}_{\boldsymbol{4}}^{\boldsymbol{2-}}\boldsymbol{+}\left( \boldsymbol{1} \right)\boldsymbol{ADP}$  $\boldsymbol{\to}\left( \boldsymbol{1} \right)\boldsymbol{C}\boldsymbol{H}_{\boldsymbol{3}}\boldsymbol{-CO}\boldsymbol{O}^{\boldsymbol{-}}\boldsymbol{+}\left( \boldsymbol{1} \right)\boldsymbol{ATP+}{\left( \boldsymbol{8} \right)\boldsymbol{Fe}}^{\boldsymbol{2+}}\boldsymbol{+}\left( \boldsymbol{8} \right) \boldsymbol{H}^{\boldsymbol{+}}$ | **-704.98** |

* See Figure 6 for corresponding reaction numbers. Mcr, methyl-coenzyme M reductase; Mtr, methyltransferase; HdrDE, membrane-bound heterodisulfide reductase; Cyt *c,* multi-heme cytochrome c; CODH/ACS, CO dehydrogenase/acetyl-CoA synthase; Pta, phosphotransacetylase; Ack, acetate kinase; Mer, F_420_-dependent methylene-H_4_MPT reductase; Mtd, F_420_-dependent methylene-H_4_MPT dehydrogenase; Mch, methenyl-H_4_MPT cyclohydrolase; Ftr, formylmethanofuran:H_4_MPT formyltransferase; Fmd, formylmethanofuran dehydrogenase; HdrA2B2C2, cytoplasmic heterodisulfide reductase; Rnf, Rnf complex.

†Standard transformed Gibbs free energy calculated using the Δ_f_G′ of metabolite formation given in Table S3.

§Calculated with published standard midpoint potentials (+772 mV for Fe3+/Fe2+ and -165 mV for MP/MPH2) using ΔG = -nFΔE and the Faraday constant of 0.09648 kJ/eV (1, 2).

¶Published elsewhere (3).

‡ Calculated with published standard midpoint potentials (+772 mV for Fe3+/Fe2+ and -520 mV for Ferredoxin*_ox_*/Ferredoxin*_red_*) using ΔG = -nFΔE and the Faraday constant of 0.09648 kJ/eV (2, 3).

1. **Tietze M, Beuchle A, Lamla I, Orth N, Dehler M, Greiner G, Beifuss U.** 2003. Redox potentials of methanophenazine and CoB-S-S-CoM, factors involved in electron transport in methanogenic archaea. ChemBioChem **4:**333-335.

2. **Thauer RK, Jungermann K, Decker K.** 1977. Energy conservation in chemotrophic anaerobic bacteria. Bacteriol Rev **41:**100-180.

3. **Catlett J, Ortiz AM, Buan N.** 2015. Rerouting cellular electron flux to increase the rate of biological methane production. Appl Environ Microbiol **81:**6528-6537.
