# Supplementary material for: A Ferredoxin- and F420H2-Dependent, Electron-Bifurcating, Heterodisulfide Reductase with Homologs in the Domains Bacteria and Archaea
Source: mBio. 2017 Feb 7;8(1):e02285-16. doi: 10.1128/mBio.02285-16 (PMC5296606; doi:10.1128/mBio.02285-16)
Supplement: TABLE S3 [file mbo001173173st3.docx]

Table S3. Gibbs free energy of metabolite formation (Δ_f_G^t^) calculated at 25˚C, pH of 7, and ionic concentration of 0.25 M.

| **Compound id** | **Compound name** | **Chemical formula (charged)** | **Δ_f_G^t^ (kJ/mol)** | **Reference** |
| --- | --- | --- | --- | --- |
| CH_3_COO | Acetate | CH_3_COO^-^ | −247.92 | (1, 2) |
| CH_3_-CO-SCoA | Acetyl-CoA | C_23_H_34_N_7_O_17_P_3_S^4-^ | −1914.82 | (1, 2) |
| CH_3_-CO-PO_4_ | Acetyl phosphate | C_2_H_3_O_5_P^2-^ | −1143.04 | (1, 2) |
| ADP | ADP | C_10_H_12_N_5_O_10_P_2_^3-^ | −1467.21 | (1, 2) |
| ATP | ATP | C_10_H_12_N_5_O_13_P_3_^4-^ | −2343.15 | (1, 2) |
| CO_2_ | Carbon dioxide | CO_2_ | −386.02 | (1, 2) |
| HS-CoA | Coenzyme A | C_21_H_32_N_7_O_16_P_3_S^4-^ | −1854.79 | (1, 2) |
| HS-CoB | Coenzyme B | C_11_H_19_NO_7_PS^3-^ | −713.09 | (1, 2) |
| F_420_ | Coenzyme F_420_ (oxidized) | C_29_H_31_N_5_O_18_P^5-^ | −1431.83 | (1, 2) |
| F_420_H_2_ | Coenzyme F_420_ (reduced) | C_29_H_34_O_18_N_5_P^4-^ | −1393.29 | (1, 2) |
| HS-CoM | Coenzyme M | C_2_H_5_O_3_S_2_^1-^ | −304.32 | (1, 2) |
| Ferredoxin_ox_ | Ferredoxin (oxidized) | Fe_8_S_8_X^3+^ | −0.81 | (3) |
| Ferredoxin_red_ | Ferredoxin (reduced) | Fe_8_S_8_X^2+^ | +38.07 | (3) |
| Fe^3+^ | Ferric iron | Fe^3+^ | −11.89 | (1, 2) |
| Fe^2+^ | Ferrous iron | Fe^2+^ | −82.11 | (1, 2) |
| CHO_2_ | Formate | CHO_2_^1-^ | −311.08 | (1, 2) |
| CHO-MF | Formyl-methanofuran | C_35_H_44_N_6_O_15_^3-^ | −458.3^*^ | [4] |
| CHO-THSPT | Formyl-THSPT | C_36_H_49_N_7_O_20_P^3-^ | −854.04 | (1, 2) |
| CoM-S-S-CoB | Heterodisulfide | C_13_H_22_NO_10_PS_3_^4-^ | −1073.76 | (1, 2) |
| CH_4_ | Methane | CH_4_ | +128.73 | (1, 2) |
| MP | Methanophenazine (ox) | C_37_N_2_O_1_H_50_ | +690.82 | (1, 2) |
| MPH_2_ | Methanophenazine (red) | C_37_N_2_O_1_H_52_ | +715.80 | (1, 2) |
| MF | Methanofuran | C_34_H_44_N_6_O_14_^2-^ | +25.86 | (1, 2) |
| CH-THSPT | Methenyl-THSPT | C_36_H_48_N_7_O_19_P^2-^ | −706.87 | (1, 2) |
| CH_3_-SCoM | Methyl-coenzyme M | C_3_H_7_O_3_S_2_^2-^ | −200.68 | (1, 2) |
| CH_3_-THSPT | Methyl-THSPT | C_36_H_51_N_7_O_19_P^3-^ | −600.75 | (1, 2) |
| CH_2_=THSPT | Methylene-THSPT | C_36_H_49_N_7_O_19_P^3-^ | −656.13 | (1, 2) |
| HPO_4_ | Phosphate | HPO_4_^2-^ | −1058.57 | (1, 2) |
| H^+^ | Proton | H^+^ | +0.08 | (1, 2) |
| THSPT | Tetrahydrosarcinapterin | C_35_H_49_N_7_O_19_P^3-^ | −733.62 | (1, 2) |
| H_2_O | Water | H_2_O | −155.65 | (1, 2) |

^*^ At pH of 7, ionic concentration of 0.1 M, and net charge of -3

1. **Alberty RA.** 1998. Calculation of standard transformed Gibbs energies and standard transformed enthalpies of biochemical reactants. Arch Biochem Biophys **353:**116-130.

2. **Jankowski MD, Henry CS, Broadbelt LJ, Hatzimanikatis V.** 2008. Group contribution method for thermodynamic analysis of complex metabolic networks. Biophys J **95:**1487-1499.

3. **Alberty RA.** 1998. Calculation of standard transformed formation properties of biochemical reactants and standard apparent reduction potentials of half reactions. Arch Biochem Biophys **358:**25-39.
