# Supplementary material for: A Ferredoxin- and F420H2-Dependent, Electron-Bifurcating, Heterodisulfide Reductase with Homologs in the Domains Bacteria and Archaea
Source: mBio. 2017 Feb 7;8(1):e02285-16. doi: 10.1128/mBio.02285-16 (PMC5296606; doi:10.1128/mBio.02285-16)
Supplement: TEXT S1 [file mbo001173173s1.docx]

**­­­­­­Materials and Methods**

**Purification of recombinant proteins.** All purification steps were performed in an anaerobic chamber (Coy Laboratory Products) containing 95% N_2_ and 5%H_2_. Cells were broken by sonication, and the lysate centrifuged anaerobically at 100,000 × *g* for 30 min to remove unbroken cells and cell debris. The supernatant was loaded onto a Ni-Sepharose column equilibrated with buffer C and washed with buffer C containing 20 mM imidazole to remove nonspecific-bound proteins. The His6-tagged proteins were eluted with buffer C containing 250 mM imidazole. The eluted proteins were further purified using a Superdex 75 gel filtration column that was developed with buffer C containing 150 mM NaCl. The peak fractions containing purified proteins were concentrated and frozen at -80℃ until use.

**Purification of CO dehydrogenase/acetyl-CoA synthase (CODH/ACS) from *Methanosarcina acetivorans*.** Crude cell-free extract of acetate-grown cells was centrifuged at 200,000 × *g* to pellet the membrane fraction. The supernatant solution (200 mg of protein in 10-ml) containing the cytoplasmic fraction was loaded onto a Q Sepharose FF column (50 ml bed volume) equilibrated with 50 mM MOPS (pH 7.0) containing 2mM DTT, 2mM dithionite, and 10% glycerol (buffer A). The column was developed with 400 ml of a 0-1.0 M NaCl linear gradient in buffer A. Each 10 ml fraction was assayed for CO-dependent reduction of methyl viologen. Ammonium sulfate (AS) was added (1.6 M, final concentration) to the pooled fractions with activity before loading onto a Phenyl-Sepharose FF column (25 ml bed volume ) equilibrated with buffer A containing 1.6 M AS. The column was developed with 200 ml of a 1.6-0 M AS decreasing linear gradient in buffer A. Peak fractions with activity were pooled and loaded onto a hydroxyapatite column (10 mL bed volume) equilibrated with 5 mM sodium phosphate (pH 7.0) containing 2mM DTT, 2mM dithionite, and 10% glycerol (buffer B). The column was developed with 100 ml of a linear gradient from 5-300 mM sodium phosphate in buffer B. The peak with greatest activity was finally purified to electrophoretic homogeneity (Fig. S2) with a Sephacryl S-300 gel filtration column developed with buffer A containing 150 mM NaCl. SDS-PAGE revealed subunit molecular masses nearly identical to the five-subunit CODH/ACS characterized from *Methanosarcina thermophila* (1).

The genome of *M. acetivorans* is annotated with duplicate CODH/ACS gene clusters (2), each encoding five subunits with nearly identical molecular masses and sequence identities greater than 98% between respective subunits. Previous proteomic analyses of acetate-grown *M. acetivorans* identified subunits CdhA, CdhB and CdhC from one cluster (MA1011-16) and CdhA, CdhB CdhC and CdhE from the other (MA3860-65) (3). Thus, it is likely that the purified CODH/ACS contained five-subunit CODH/ACS complexes encoded by both gene clusters.

**Enzyme assays.** Heterodisulfide reductase activity was performed as described elsewhere with the following modifications (4). Forward activity was assayed by using reduced MV as an electron donor. After addition of 2 mM MV, the cuvettes were titrated with 90 mM titanium (III) citrate until the absorbance at 604 nm reached a value of 1.5. Subsequently, 50 ug protein was added and the reaction was started by addition of CoM-S-S-CoB to a final concentration of 0.28 mM. Reverse activity was assayed by monitoring reduction of 0.15 mM methylene blue with 0.5-5 mM HSCoM and HSCoB. The reaction was started by addition of 50 ug of enzyme. A unit of enzyme activity (U) is the amount catalyzing the reduction or formation of 1 umol CoM-S-S-CoB per min.

Fdx:CoMS-SCoB oxidoreductase assays shown in Table 1 were performed anaerobically in an atmosphere of either N_2_ or CO. Cell-free extract of acetate-grown *M. acetivorans* was prepared as previously described (5). The membrane and cytoplasmic fractions were prepared by discontinuous sucrose gradient ultracentrifugation as previously described (5). The cytoplasmic fraction was further centrifuged at 200,000 x *g* to remove any residual membranes. Fdx:CoMS-SCoB oxidoreductase activity was assayed with two systems for regenerating reduced Fdx purified from *M. acetivorans* as previously described (5). The FNR (***F***dx-***N***ADP^+^ ***r***eductase) system utilized the plant enzyme catalyzing oxidation of NADPH and reduction of Fdx. The CO dehydrogenase (CODH) system utilized the αε-subunit component of the five-subunit CODH/ACS purified from acetate-grown *M. acetivorans* as previously described (5). For the FNR system, the reaction mixture (222 μl) contained 18 mM NADPH, 2 μM Fdx, 0.9 mM CoMS-SCoB and either the cytoplasmic fraction (159 μg protein) or cell extract (166.8 μg protein), or the membrane fraction (106.8 μg protein) contained in 50 mM MOPS (pH 6.8). FNR (4.0 μg) was added to initiate the reaction. For the CODH system, the reaction mixture (200 μl) contained 5 μM Fdx, 2.24 μg CODH and 320 μM CoMS-SCoB in 50 mM MOPS (pH 6.8). The atmosphere was 100% CO. The cytoplasmic (75 μg protein) or membrane fraction (178 μg protein), or cell lysate (111 μg protein), was added to initiate the reaction. For both assays, aliquots of the reaction mixture were removed at time intervals and the content of free thiols determined with Ellman’s reagent as described elsewhere (6).

**REFERENCES**

1. **Abbanat DR, Ferry JG.** 1991. Resolution of component proteins in an enzyme complex from *Methanosarcina thermophila* catalyzing the synthesis or cleavage of acetyl-CoA. Proc Natl Acad Sci USA **88:**3272-3276.

2. **Galagan JE, Nusbaum C, Roy A, Endrizzi MG, Macdonald P, FitzHugh W, Calvo S, Engels R, Smirnov S, Atnoor D, Brown A, Allen N, Naylor J, Stange-Thomann N, DeArellano K, Johnson R, Linton L, McEwan P, McKernan K, Talamas J, Tirrell A, Ye W, Zimmer A, Barber RD, Cann I, Graham DE, Grahame DA, Guss AM, Hedderich R, Ingram-Smith C, Kuettner HC, Krzycki JA, Leigh JA, Li W, Liu J, Mukhopadhyay B, Reeve JN, Smith K, Springer TA, Umayam LA, White O, White RH, de Macario EC, Ferry JG, Jarrell KF, Jing H, Macario AJ, Paulsen I, Pritchett M, Sowers KR, et al.** 2002. The genome of *M. acetivorans* reveals extensive metabolic and physiological diversity. Genome Res **12:**532-542.

3. **Li L, Li Q, Rohlin L, Kim U, Salmon K, Rejtar T, Gunsalus RP, Karger BL, Ferry JG.** 2007. Quantitative proteomic and microarray analysis of the archaeon *Methanosarcina acetivorans* grown with acetate *versus* methanol. J Proteome Res **6:**759-771.

4. **Hedderich R, Berkessel A, Thauer RK.** 1989. Catalytic properties of the heterodisulfide reductase involved in the final step of methanogenesis. FEBS Lett **255:**67-71.

5. **Wang M, Tomb JF, Ferry JG.** 2011. Electron transport in acetate-grown *Methanosarcina acetivorans*. BMC Microbiol **11:**165.

6. **Ellman GL.** 1958. A colorimetric method for determining low concentrations of mercaptans. Arch Biochem Biophys **74:**443-450.

7. **Stojanowic A, Mander GJ, Duin EC, Hedderich R.** 2003. Physiological role of the F_420_-non-reducing hydrogenase (Mvh) from *Methanothermobacter marburgensis*. Arch Microbiol **180:**194-203.
